# Crude oil as a microbial seed bank with unexpected functional potentials

**DOI:** 10.1038/srep16057

**Published:** 2015-11-03

**Authors:** Man Cai, Yong Nie, Chang-Qiao Chi, Yue-Qin Tang, Yan Li, Xing-Biao Wang, Ze-Shen Liu, Yunfeng Yang, Jizhong Zhou, Xiao-Lei Wu

**Affiliations:** 1 Peking University, College of Engineering, Beijing, 100871, China; 2 Sichuan University, College of Architecture and Environment, Chengdu, 610065, China; 3 Tsinghua University, School of Environment, State Key Joint Laboratory of Environment Simulation and Pollution Control, Beijing, 100084, China

## Abstract

It was widely believed that oil is a harsh habitat for microbes because of its high toxicity and hydrophobicity. However, accumulating evidence has revealed the presence of live microbes in crude oil. Therefore, it’s of value to conduct an in-depth investigation on microbial communities in crude oil. To this end, microorganisms in oil and water phases were collected from four oil-well production mixtures in Qinghai Oilfield, China, and analyzed for their taxonomic and functional compositions via pyrosequencing and GeoChip, respectively. Hierarchical clustering of 16S rRNA gene sequences and functional genes clearly separated crude oil and water phases, suggestive of distinct taxonomic and functional gene compositions between crude oil and water phases. Unexpectedly, *Pseudomonas* dominated oil phase where diverse functional gene groups were identified, which significantly differed from those in the corresponding water phases. Meanwhile, most functional genes were significantly more abundant in oil phase, which was consistent with their important roles in facilitating survival of their host organisms in crude oil. These findings provide strong evidence that crude oil could be a “seed bank” of functional microorganisms with rich functional potentials. This offers novel insights for industrial applications of microbial-enhanced oil recovery and bioremediation of petroleum-polluted environments.

Oil is modern industry’s lifeblood, but it is also a source of environmental pollution worldwide. In the oil recovery industry, water flooding, i.e., pressurized water being pumped into oil-bearing rock strata to push oil out of the reservoirs, is widely used^1^. However, water flooding has its production limit at ~30% oil recovery, leaving a significant amount of oil untapped^2^. Microbial enhanced oil recovery (MEOR) is a cost-effective technique to recover the residual oil^3^. Generally, microorganisms or nutrients are injected into the oil reservoirs to propagate microorganisms in deep subsurface, which resulted in higher microbial activities that improve oil fluidity and recovery, as file tests in Romashkinskoe Oilfield^4^ and Dagang Oilfield^5^. Therefore, understanding the microbial processes in deep oil reservoirs is important for the development of MEOR. Because it is extremely difficult to aseptically sample the oil-bearing rock cores, the injection water and production mixtures are often sampled and analyzed using both culture-dependent and -independent methods to assess the microbial community of an oil reservoir. For example, analysis of the 16S rRNA gene fingerprints using a culture-independent clone library^6^,^7^,^8^, denaturing gradient gel electrophoresis (DGGE)^9^,^10^, terminal restriction fragment polymorphism (T-RFLP)^11^, and pyrosequencing^1^,^12^ revealed that the major bacteria in oilfields worldwide included *Proteobacteria*, *Firmicutes*, *Bacteroidetes*, and *Planctomycetes*, though their relative abundances vary among oilfields^1^,^13^. In addition, significant differences in bacterial composition have been detected between injected water and production mixtures in most oilfields. For example, only 2.86% of bacterial operational taxonomic units (OTUs) and 15.15% of archaeal OTUs were shared between injection water and the production mixture in the Gudao Oilfield, suggesting that injected water had only a minor influence on the microbial community indigenous to the oil containing strata^10^,^11^. Although diversities of several functional genes were investigated in some oilfields, such as the methyl coenzyme M reductase genes (*mcr*)^14^ and dissimilatory sulfite reductase genes (*dsr*)^15^, community-scale functional gene diversity has not been assessed thoroughly. Therefore, the overall functional potential of microbes from these oilfields remains unclear.

It is widely believed that oil is not a preferred habitat for microbes because of its potential toxicity and high hydrophobicity^16^. However, recent studies have shown that microorganisms including *Acinetobacter*, *Propionibacterium*, *Sphingobium*, *Bacillales*, *Burkholderia*, and *Brevundimonas* are present in crude oil phase^17^,^18^. In a recent study, we observed alive microbial cells in crude oil, which could be stimulated to bloom by introduction of other bacteria^19^. In addition, different microbial communities from the oil and water phases of the same oil production mixture were detected in a high-temperature oilfield in Japan^20^ and a mesothermic oilfield in Canada^12^. Hydrogen- and/or hydrocarbon-utilizing bacteria and archaea preferentially attached to the crude oil phase, whereas *Deltaproteobacteria* were the main microbial group identified in the water phase^12^. The detection of these microorganisms in crude oil is changing our understanding of microorganisms, as well as crude oil itself. However, it is questionable whether highly hydrophobic oil accommodates microorganisms ubiquitously, and whether functional potentials of the microorganisms residing in crude oil are distinct from those in corresponding water phase. Moreover, because crude oil microenvironments in oil reservoirs are thought to be hardly disturbed by the surface water^21^, it is of much interest whether the microbes residing in the subterranean oil phase evolve in a way different from those in the water phase or other environments. In addition, it is typical to detect several oil-bearing strata with different depth in an oilfield. Do these different crude oils accommodate different functional potentials due to variations in, pressure and temperature? If the answers to these questions are yes, it would be of great interest to examine whether crude oil itself is a reservoir of rare species that can potentially expand rapidly under suitable conditions, which is defined as a “seed bank” - a reservoir of dormant individuals that can expand rapidly when conditions are right^22^,^23^. This reservoir of functional microorganisms might play important roles in the natural oil attenuation process during the long history of oil reservoir formation. Moreover, such an investigation could be important for developing technologies such as MEOR and bioremediation of oil-polluted environments.

Here, we used high-throughput pyrosequencing and a microarray-based metagenomic tool GeoChip 4.0^24^ to investigate the microbial communities between oil and water phases which were sampled from four oil-containing rock strata with different depths (temperatures) and groundwater salinities at the Qinghai Oilfield (Fig. 1). Unexpectedly, highly diverse functional groups in microorganisms associated with the oil phase were detected, and significant differences between the crude oil- and water-related microbial communities were observed. Furthermore, microbial communities in oil phases from different rock strata were taxonomically similar, but their functional potentials differed significantly. Because of the diverse functional potentials, crude oil itself could be a “seed bank” of functional microorganisms for elucidating crude oil-related natural microbial processes and developing new oil recovery technologies.

## Results

### Geochemical characteristics of samples

**Figure 1:**
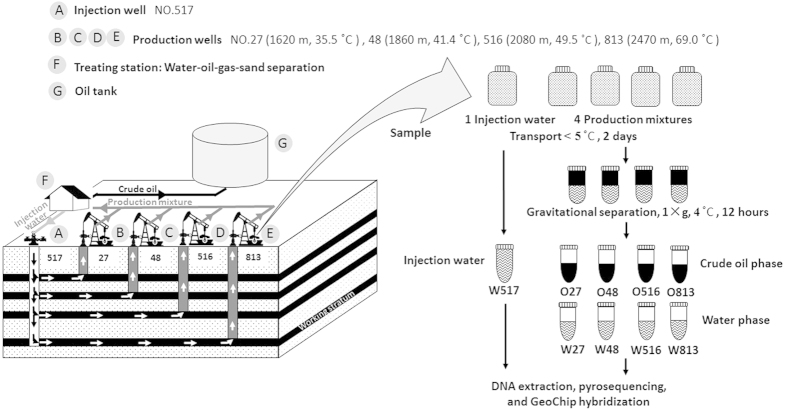
Outline of sampling and sample management. The model of four oil-containing rock strata with different depths and proceeding of oil extraction in Qinghai Oilfield, as well as the sample management in lab after sampled from oilfield. All drawings were drew by Man Cai.

Chemical analyses revealed that concentrations of the total dissolved solids (TDS) of the water (salinity) from the oilfield blocks ranged from 33 to 98 g·L^−1^ (Table S1). TDS differed substantially between water samples from different layers of oil-containing rock in oil reservoir (oil strata) where the oil was extracted (Fig. 1). Because the injection water was the same amongst the reservoirs, so the differences of TDS concentrations were reservoir-attributable changes, suggesting that the process of water flooding for 10 years had a minor influence on ground water in different oil strata, which was in consistent with previous results^10^,^25^. As the oil-containing stratum temperature/well depth increased, weight ratios of aromatic hydrocarbon (ARH) decreased and aliphatic hydrocarbons (ALH) increased (Table S2).

### Functional distribution of microbial communities

**Figure 2:**
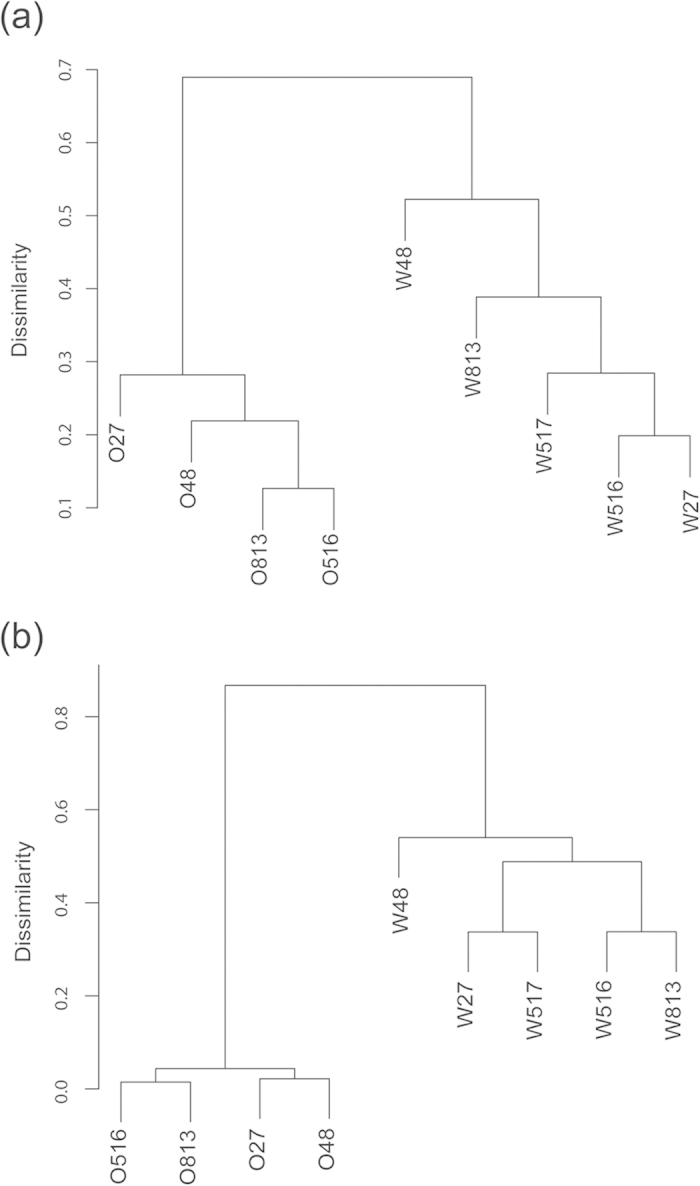
Hierarchical clustering analysis of functional genes and microbial communities. Hierarchical clustering analysis of all samples based on relative signal abundances of functional genes in GeoChip hybridization (**a**) and on the abundance of OTUs obtained by pyrosequencing (**b**).

GeoChip 4.0 contains approximately 82,000 probes for targeting >141,995 coding sequences from 410 functional gene families related to microbial carbon (C), nitrogen (N), sulphur (S), and phosphorus (P) cycling, energy metabolism, antibiotic resistance, metal resistance/reduction, organic remediation, stress responses, bacteriophage and virulence^26^. GeoChip 4.0 hybridization experiments led to detection of total of 35,017 genes from 397 gene families in the nine samples. Hierarchical clustering analysis based on functional gene abundances showed that the samples clustered into two groups. One group contained all four samples from oil phase, and another group contained all four samples from water phase, which suggested that microbial functional diversity was different between the oil and water phases (Fig. 2a).

### Functional gene distribution differences between the oil and water phases

Unexpectedly, alpha diversities of detected functional genes, as examined by the Shannon-Weiner index, were significantly (P < 0.05) higher in the oil phase (9.621 ± 0.074, mean ± SD) than those in water phase (9.438 ± 0.255) from the same production well (Table S3). Beta diversities of functional genes between oil phases (0.261 ± 0.045) were lower than those between water phases (0.361 ± 0.077), and between oil phases and water phases (0.360 ± 0.096) (Table S4). The significant difference between the oil and water phases in functional composition was demonstrated by three non-parametric multivariate statistical tests (MRPP, ANOSIM and Adonis; Table S5).

**Figure 3:**
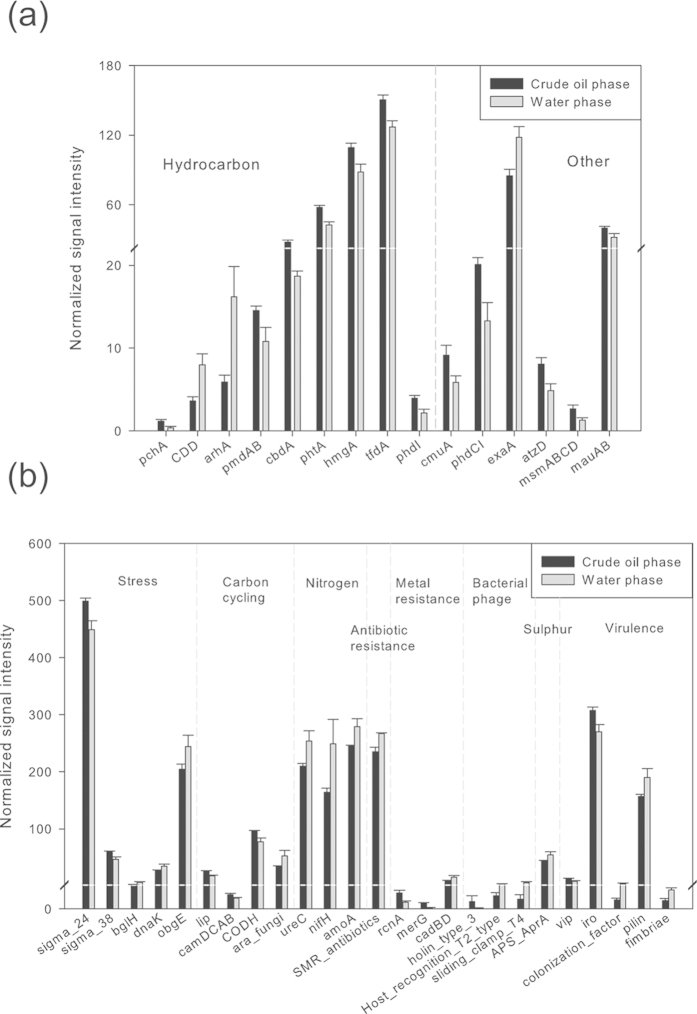
Significant genes in different living activities between the crude oil and water phase samples. The normalized signal intensity of the significant (P < 0.05) gene in the organic degradation category (**a**) and stress regulation, carbon cycling, nitrogen and sulfur metabolism, antibiotic resistance, metal resistance, bacteriophage, and virulence categories (**b**) between the crude oil phase and water phase samples. The signal intensity for the gene was the average of the total signal intensity from all four samples of the same phase.

Organic compound degradation genes were highly abundant, with significantly (P < 0.05) more abundance in crude oil than in the water phase (Fig. 3a). For example, the average total abundance of *phtA*, *hmgA*, and *pmdAB* genes encoding the first enzymes in the polycyclic aromatic hydrocarbon catabolic pathway that converts hydrocarbons into aromatic carboxylic acids were significantly (P < 0.05) higher in the crude oil phase (Fig. 3a). The carbon cycle genes *lip*, *camDCAB* and *CODH*, which encode manganese peroxidase for lignin degradation, camphor monooxygenase, and carbon monoxide oxidase, respectively, were also detected at a significantly (P < 0.05) higher abundance in the oil phase (Fig. 3b). By contrast, some genes such as the alkane 1-monoxygenase gene (*alkB*), which encodes a key enzyme in aerobic metabolism of aliphatic hydrocarbons, showed similar abundance in the water and oil phases.

Of the stress response genes, a group of 711 σ^24^ genes and 93 σ^38^ genes was found to be significantly (P < 0.05) more abundant in crude oil than the 685 σ^24^ genes and 75 σ^38^ genes detected in the water phase (Fig. 3b). Conversely, three other stress-related genes, *bglH* (encoding a beta-glucosidase), *dnaK* (encoding a heat shock protein), and *obgE* (encoding a GTPase), had significantly (P < 0.05) higher abundance in water than in the crude oil phase (Fig. 3b). Furthermore, genes involved in nitrogen and sulfur metabolism (including *ureC*, *nifH*, *amoA*, and *APS_AprA*, which encode urease for ammonification, nitrogenase for nitrogen fixation, ammonia monooxygenase for ammonia oxidation, and adenylylsulfate reductase for sulfate reduction, respectively), showed significantly (P < 0.05) higher abundance in water than in the oil phase (Fig. 3b). A total of 38 bacteriophage genes were detected, of which the abundances of three genes varied significantly (P < 0.05) between the crude oil and water phases: *holin_type_3* (higher abundance in the oil phase), *host_recognition_T2_type* (higher abundance in the water phase), and *sliding_clamp_T4* (higher abundance in the water phase), which were related to bacterial lysis, host recognition, and replication, respectively (Fig. 3b).

### Differences in functional gene distribution among oil samples

As the temperature and depth of oil-containing stratum decreased (Fig. 1), the relative abundance of genes related to the degradation of aromatic compounds, carbon cycling, and metabolism of other organic compounds increased concomitantly (by 2.2%, 3.1%, and 15.3%, respectively) (Table S6). For example, the abundance of *nahA*, *HBH* and *pobA* genes involved in the aerobic degradation of benzoate, a common central intermediate in the metabolism of aromatic compounds, increased. The abundance of *hmgA*, which encodes phthalate dioxygenase, also increased (Table S7). In contrast, the increase/decrease of genes abundances was not found among the five water samples as the temperature of oil-containing stratum decreased (Table S6).

Similarly, the relative abundance of genes associated with the stress response, antibiotic resistance, and sulfur metabolism decreased (1.6%, 3.1%, and 1.6%, respectively) with temperature/depth (Table S6). For example, 133 genes, including two *dnaK* genes, which encode the molecular chaperone HSP70 and up-regulated upon heat shock^27^, increased with temperature (Table S8), whereas the abundance of 75 genes, including *hrcA*, which encodes a heat-inducible transcriptional repressor of bacterial heat shock genes^28^, decreased (Table S9).

### Taxonomic distribution of the bacterial community

A total of 41,792 valid sequences were retrieved from pyrosequencing analysis, including 4,613–6,612 sequences from each oil-phase sample that were assigned to 152–186 bacterial OTUs (>97%), and 2,837–4,154 sequences from each water-phase sample that were assigned to 138–246 bacterial OTUs (Table S10). The coverage values and Chao1 indices (219–452) showed sequencing saturation, thus the number of sequences was adequate to represent the bacterial community in each sample. The Shannon-Wiener indices indicated that bacterial communities were less diverse (1.605 ± 0.093) in oil phases than in water phases (3.246 ± 0.321, mean ± SD) (Table S10). Hierarchical clustering analysis of OTU abundance clearly separated the samples into two clusters of crude oil and water, suggesting that these microbial communities were markedly different (Fig. 2b). In addition, OTUs common among the injection, oil, and water phases were limited (Fig. S1). Furthermore, MRPP, ANOSIM and Adonis analyses showed the significant difference between the oil and water phases in taxonomic composition, in accordance with the results based on functional gene composition (Table S5).

**Figure 4:**
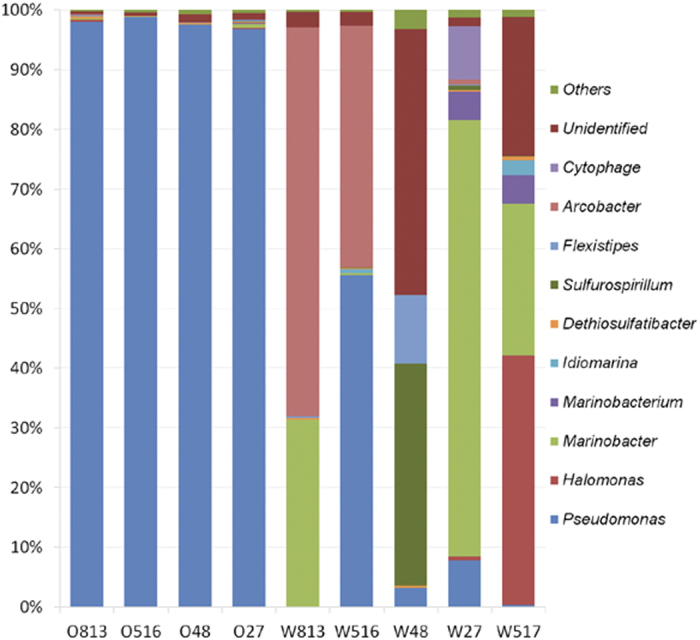
Relative abundance of major bacterial genera detected in each samples. The relative abundance based on OTU numbers of different genera by pyrosequencing from each crude oil and water phases sample.

A total of nine phyla were retrieved from the 41,792 sequences, of which 361 sequences could not be assigned to any known bacterial phyla. *Proteobacteria* was the most abundant phylum in all the oil phases, ranging from 97.86% to 99.02%. By contrast, *Firmicutes* and *Bacteroidetes* accounted for 9.20% and 17.31% of the total sequences in the W27 and W517 (injection) water samples, respectively. *Chloroflexi* was detected in only W516 (0.03%), whereas *Deferribacteres* (11.60%) and an abundant, unidentified bacteria (10.86%) were present in W48 (Table S11). Specifically, *Gammaproteobacteria* dominated the crude oil phase and both *Gammaproteobacteria* and *Epsilonproteobacteria* were generally dominant in the water phases, but with varying abundances (Fig. 4, Table S12).

At the genus level, 50 genera were retrieved from all the samples. Among these genera, 33 and 31 known genera were retrieved from the oil samples and the water samples, respectively. *Pseudomonas* was abundant in the four oil samples, accounting for 96.84–98.87% of the total sequences in each sample (Fig. 4, Table S12). However, it was present at much lower levels in the water samples with a relative abundance of 0.00–7.86%, except for 55.57% in W516. *Acinetobacter* and *Dethiosulfatibacter* were also common in oil phases, but at a very low abundance (Fig. 4, Table S12). Finally, *Marinobacter*, *Halomonas*, and *Flexistipes* were detected in three of the oil samples. The bacteria in water samples were more diverse, without a common dominant representative. The bacteria common to the five water samples were *Sulfurospirillum* (0.03–37.19%) and *Dethiosulfatibacter* (0.16–0.63%) (Fig. 4, Table S12). The major bacterial genera in W813, W516, W48, and W27 were *Arcobacter* (65.13%) and *Marinobacter* (32.16%); *Pseudomonas* (55.57%) and *Arcobacter* (40.50%); *Sulfurospirillum* (37.19%) and *Flexistipes* (11.56%); and *Marinobacter* (73.12%), *Cytophage* (8.92%), *Pseudomonas* (7.86%), and *Marinobacterium* (4.74%), respectively. In the injection water (W517), *Halomonas* (41.86%), *Marinobacter* (25.35%), *Marinobacterium* (4.84%), and *Idiomarina* (2.50%) were the major bacterial genera, accounting for 74.55% of the total number sequences.

**Figure 5:**
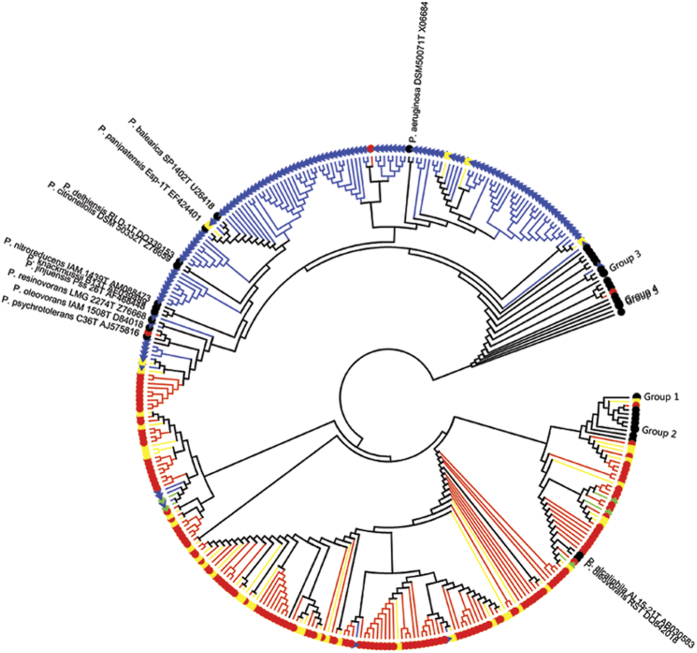
Phylogenetic relationships of *Pseudomonas* in crude oil and water phases. Circle topology tree of the phylogenetic relationships between the 308 bacterial 16S rRNA gene sequences obtained from pyrosequencing OTUs in this study and 125 valid *Pseudomonas* species strains from RDP. Bar, 1nt substitution per 100 nt. 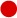 OTUs only from crude oil phases; 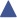 OTUs only from oil production water phases; 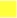 OTUs both from oil and production water phases; 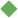 OTUs both from injection water and oil and production water phases; no OTUs only from injection water; 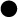 valid *Pseudomonas* species (all the names of *Pseudomonas* species strains were list in Table S13).

Although *Pseudomonas* was detected in both the water and oil phases, detected species were distinct between the water and oil phases (Fig. 5), i.e., those from the oil phase clustered with *P*. *oleovorans* and *P*. *alcaliphila*, while those from the water phase clustered with *P*. *aeruginosa*, *P*. *balearica*, and *P*. *citronellolis*.

### Association between microbial community and geochemical characteristics

**Figure 6:**
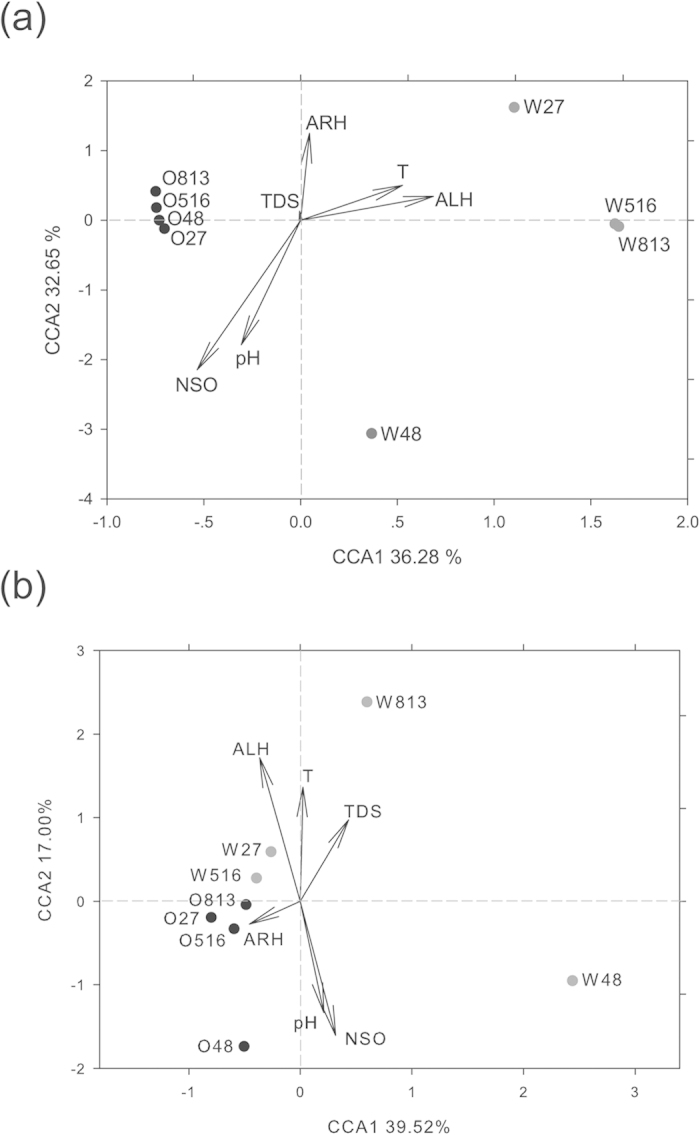
Canonical correspondence analysis (CCA) of significant geochemical variables and microbial communities from all production well samples. (**a**) CCA of significant geochemical variables (arrows) and relative abundances of bacterial OTUs (symbols). (**b**) CCA of significant geochemical variables (arrows) and relative abundance of functional genes from GeoChip hybridization signals (symbols). Variables: aliphatic hydrocarbons (ALH), aromatic hydrocarbons (ARH), polar fraction with heteroatoms nitrogen, sulfur, and oxygen (NSO) from crude oil, total dissolved solid (TDS) of production water, pH value, and bottom hole temperature of production wells (T).

The links between environmental conditions and microbial taxonomic structures and functional gene compositions were evaluated using canonical correspondence analysis (CCA) (Fig. 6a,b). The key geochemical parameters for both water and crude oil phases included TDS, pH, ALH, ARH, polar fraction with heteroatoms nitrogen, sulfur, and oxygen (NSO), and oil-containing stratum temperature (T), which were selected after various pre-evaluations. Similar to the results of hierarchical clustering using the functional gene abundances or OTU numbers, all of the oil phases were clustered and well separated from the water samples. In addition, the four water phases were separated into different quadrants.

For taxonomic composition, the first axis positively correlated with T and ALH, whereas the second axis positively correlated with ARH and negatively correlated with pH and NSO (Fig. 6a). For functional gene composition, the first axis negatively correlated with ARH, whereas the second axis positively correlated with ALH and T, and negatively correlated with NSO and pH (Fig. 6b).

## Discussion

Despite the marked significance in understanding microorganisms mediated processes in an oilfield, microorganisms in crude oil, their functional profiles and their differences between oil and water phases remain unclear. To our knowledge, this is the first comprehensive study to investigate and compare microbial communities in crude oil and water phases at both the taxonomic and functional gene levels. Our results revealed that microorganisms in crude oils were taxonomically narrow but extremely diverse in functional potentials (Table S3 and S10, and Figs 3 and 5).

Although the microbial communities in the oil phase were dominated by *Pseudomonas*, sequencing results showed that there existed a rare biosphere, which accounted for less than 3.16% of the total OTUs in the oil samples (Table S12). These OTUs spanned from 33 known genera belonging to phyla *Proteobacteria*, *Firmicutes*, *Deferribacteres*, and *Bacteroidetes*. In contrast, although the bacterial communities in the water phase are more diverse than those in the oil phase, the OTUs in the water phase could be only assigned into 31 known genera, which was even less than those in the oil phase. The genera with high abundances such as *Arcobater*, *Marinobacter* and *Sulfurospirillum* in the water phase could also be found in the oil phase. Moreover, dominant taxa in the water phases were different, which might be caused by the different temperatures, depths and pH, suggesting a rapid growth of these taxa in the water phase under the appropriate environmental conditions. The overlap of taxa also indicated the dispersal of microorganisms between the oil and water phase, although the direction of dispersal seemed to be unclear. However, at least for *Pseudomonas*, most of the shared OTUs by both oil and water phases were embedded in the OTUs clusters of oil phase (Fig. 5), which suggested that the dispersal of these OTUs was most likely from the oil phase to the water phase. *Pseudomonas*, usually a mesophilic microorganisms, has been detected more and more from high temperature oilfields^11^,^29^,^30^,^31^, and in our previous investigation, we also detected *Pseudomonas* from autoclaved crude oil^19^. It may be related to the known ability of these organisms to form biofilms^32^,^33^ thus giving them a competitive advantage in high-temperature anhydrous environments. Recent study also suggested that microorganisms in oil lived in minuscule water droplets which provided closed microenvironments to protect them^21^. These results supported that the crude oil could act as a microbial “seed bank” and influence the microbial communities in oil reservoirs. As the sequencing depth is expected to increase due to rapidly expanding sequencing capacity, it is very likely that more taxa will be found in the oil phase, in accordance with relatively high abundance of functional genes found in the oil phase. Because GeoChip is sensitive in detecting genes of low abundance^34^, extremely diverse gene functions were detected, suggesting that crude oil harbors a microbial seed bank with rich functional potential.

Several studies have demonstrated the taxonomic differences between the oil and water phases^12^,^20^. In this study, both GeoChip and pyrosequencing analyses revealed a significant (P < 0.05) difference in microbial communities between the crude oil and water phases, even though they were from the same oil production mixture (Figs 2 and 4; Tables S3 and S4). However, little was known why there were significant taxonomic differences between the oil and water phases. This question could also be explained as what conditions in the water phase promoted the growth or changed the relative abundance of taxa in the communities, if the crude oil served as a “seed bank”. It was possible that different microbial communities were caused by dispersal or growth of rare and dominant taxa from the microbial “seed bank”, which changed the abundance of taxa rather than the presence or absence of different taxa. So different environmental filtering might be important in shaping the communities.

The microorganisms in oil and water phases displayed different correlations with the environmental conditions. CCA analysis revealed that the microbial functional community was closely related to the crude oil components (ARH, ALH, and NSO), the stratum temperature, and pH (Fig. 6b). It is well established that crude oil contains more than 3,000 organic compounds^35^, which provides a wide variety of niches for microbial communities and allows for gene drift among microbial groups^36^. However, high toxicity and hydrophobicity impose strong natural selection on these microorganisms^37^,^38^, resulting in highly uneven microbial communities that could serve as a microbial seed bank. Remarkably, our previous results showed that *Pseudomonas*, *Bacillus*, and *Alcaligenes* survived autoclaving in crude oil and could be stimulated by *Dietzia* and *Acinetobacter* strains^19^. *Pseudomonas* and *Bacillus* eventually outcompeted other bacteria and played the most important roles in crude oil degradation.

The oil phase contained a higher abundance of genes involved in aromatic degradation and carbon cycling compared to the water phase. Crude oil contains more carbon sources such as alkanes and aromatic hydrocarbons than the water phase, though these substrates are hydrophobic^12^. Of the bacteria detected by pyrosequencing, *Pseudomonas* has a known ability to degrade oil aromatic compounds using the enzymes encoded by *phtA*, *hmgA*, and *pmdAB*^39^,^40^,^41^. Moreover, microorganisms could produce surface-active materials and changing their cell surface hydrophobic activities to access hydrophobic oil constituents. Some *Pseudomonas* spp., e.g., *P. aeruginosa*, can produce biosurfactants such as rhamnolipids^42^ that help access crude oil. This allowed *Pseudomonas* to attach to crude oil and utilize the aromatic carbon sources, explaining the significantly (P < 0.05) higher abundance of aromatic degradation-related genes in the oil phase (Fig. 3a). In contrast to the oil phase, the water phase contained more oxygenated and sulfonated molecules from biodegradation of crude oil, such as carboxylic acids^21^. As the results, it was unsurprising that we found many microorganism that were not oil degraders in the water phase.

The different environmental conditions could also explain the differences among the water samples from four wells. The water phase samples from the Qinghai Oilfield had high TDS contents ranging from 33–98 g·L^−1^, which promotes the growth of halophilic organisms such as *Halomonas* and *Marinobacter*^43^,^44^. Consistently, non-halophilic *Pseudomonas* was the most abundant microbial group in W516, which had the lowest salinity/TDS ratio (33.2 g·L^−1^). However, significantly (P < 0.05) more genes related to stress response, e.g., σ^24^ and σ^28^, were found in the oil phase (Fig. 3b), which cannot be attributed to salinity. Rather, high hydrophobicity of crude oil causes high osmotic pressure, which might lead to more stress response genes in oil phase.

It was interesting that more abundant host recognition- and replication-related bacteriophage genes, such as *host_recognition_T2_type* and *sliding_clamp_T4*, were detected in the water phase than those in the oil phase (Fig. 3b). Because the water samples were filtered with 0.22-μm membranes, it could be imagined that free phages could hardly remain in the samples. The detection of these phage-related genes might be possibly from the microbial genomes, which might be the traits of the phage infection events during the evolution. Recent study suggested that microorganisms in oil lived in minuscule water droplets, and the high salinity and water-stable isotopes of the droplets indicated a deep subsurface origin^21^, raising a possibility that water droplets and their microbial habitants might originate from the ancient seawater or brines. The water droplets provided closed environments and protected the migration of microorganisms from the outside intrusions such as water injection to the oil embedded water droplets. In contrast, the production water was more prone to dispersal and migration and could be influenced by the injection water or the surface water^45^, as well as by the oil phase in the oil-water transition zone. The more closed microenvironments in the oil phase might protect the inner microbes from phage infections, which could explain the higher abundance of bacteriophage genes in the water phase. This discovery was also consistent with the important role of phage in phage-driven microbial life cycles under the condition of oil-polluted water treatment^46^. However, further investigations are still needed to clarify the hypotheses.

In summary, despite the wide belief that oil is a harsh habitat, here we show that oil harbors unexpectedly rich microbial functional potentials. This is the first study to demonstrate that crude oil could be a “seed bank” of functional microorganisms enriched for hydrocarbon degradation-related capacities. Therefore, a microbial community related to crude oil should be considered when evaluating the natural crude oil attenuation process, as well as when developing and applying technologies for bioremediation and MEOR.

## Methods

### Site and sampling

We collected the injection water and production mixture samples from Yue-II block of the Qinghai Oilfield, which is located in the Tibetan Plateau, northwestern China. This block had been subjected to water flooding by the local oil company since 1999. In this work, we selected four oil wells (numbers 813, 516, 48, and 27) which were connected to four different oil-containing rock strata with different depth, temperature, and salinity (Table S1 and S2), as well as their common injection well (number 517) for sampling (Fig. 1). The distances between the four production wells and the injection well ranged from 0.2 to 0.5 km. For each oil well or injection well, we collected production mixtures or the injection water from the sampling valve on the well head into sterile bottles, and filled the bottles to exclude oxygen. Then we sealed the bottles with sterile screw caps and sent them immediately back to the laboratory within 48 h below 5 °C. After receiving, we settled all the bottles filled with production mixtures with gravitational precipitation^12^ for 12 h at 4 °C to facilitate separation of the oil/water phases. Subsequently, we collected the production water layer and oil layer from each bottle as four water phase samples (W813, W516, W48, and W27, corresponding to the four wells), and oil phase samples (O813, O516, O48, and O27), respectively. The injection water sample (W517) was subjected to direct analysis.

### Chemical analysis

Four sub-fractions comprising aliphatic hydrocarbons (ALH), aromatic hydrocarbons (ARH), a polar fraction with nitrogen-, sulfur- and oxygen-containing compounds (NSO), and asphaltenes (ASP) were separated from the four crude oil samples using liquid-solid chromatography^47^. We weighed them to calculate the weight ratios of the fractions, then measured contents of the different elements in the crude oil using a Vario EL III elemental analyzer (Elementar Analysensysteme GmbH, Germany) for carbon and hydrogen, and a multi EA3100 elemental analyzer (Analytik Jena AG, Germany) for sulfur and nitrogen. After filtration through 0.22 μm cellulose ester membrane filters, we measured the concentrations of Na^+^, K^+^, Mg^2+^, Ca^2+^, Cl^−^, and SO_4_^2−^ in the water samples using cation and anion columns (IC-C4 and IC-A3[s], Shimadzu, Japan) in PIA-1000 ion analyzer (Shimadzu, Japan), according to the manufacturer’s instructions. Then total dissolved solids (TDS) was calculated as sum of concentrations of all the tested ions.

### DNA extraction, GeoChip hybridization, and pyrosequencing

After separation, we collected the microbial cells of the injection water and the four water phase samples by filtering them through 0.22-μm cellulose ester membrane filters. Meanwhile, we collected the microbial cells from the four oil-phase samples as described previously^18^ with several modifications. Briefly, we mixed 15 mL of the oil with 15 mL of sterile isooctane (2, 2, 4-trimethylpentane) and settled the mixture overnight at 4 °C. Then we vortexed (1000 rpm) the mixture for 5 min and centrifuged at 5,000 × *g* for 1 h at 4 °C. After resuspension of the precipitants in 20 mL isooctane, centrifuged at 5,000 × *g* three times, and lyophilized. We extracted and purified microbial genomic DNA from each of these portions as previously described^48^,^49^. Because the samples were kept at low temperature for less than 12 hours (overnight), the nutrients in the samples, such as nitrogen and phosphorus, were low, and the hydrocarbons were not easily degradable as carbon sources at such low temperature and short time, we don’t think that there were major changes in the microbial communities by isooctane addition and subsequent steps. DNA quality and quantity were measured using NanoDrop ND-1000 Spectrophotometer (NanoDrop Technologies Inc., Wilmington, DE, USA) and with PicoGreen using a FLUOstar Optima (BMG Labtech, Jena, Germany), respectively^26^. For microbial functional diversity study, we used GeoChip 4.0 to dissect the microbial community functional gene composition as described elsewhere^50^. We labeled DNA samples with the fluorescent dye Cy-3 using a random priming method and purified using the QIA quick purification kit (Qiagen, Valencia, CA, USA). And then dried DNA in a SpeedVac (ThermoSavant, Milford, MA, USA) at 45 °C for 45 min. The hybridization was carried out at 42 °C for 16 h on a MAUI hybridization station (BioMicro, Salt Lake City, UT, USA). After purification, we scanned GeoChip microarrays using a NimbleGen MS200 scanner (Roche, Madison, WI, USA) at 633 nm using a laser power and photomultiplier tube gain of 100% and 75%, respectively^26^. For microbial composition study, we used purified DNA to amplify the bacterial hypervariable V3 region of the 16S rRNA gene (at *Escherichia coli* 16S rRNA gene position 341–534 bp) to construct a community library using tag pyrosequencing (for more details, please see Supplementary Methods).

### Statistical analysis

For GeoChip analysis, we quantified and processed signal intensities using the data analysis pipeline as previously described^27^,^49^,^51^. After removing the poor quality data with ratio of signal to noise lower than 2.0, we normalized the signal intensity of each probe as described previously^49^. These normalized data were relative functional gene abundances or relative signal intensities which were then used for further analysis. We examined beta diversity to analyze the dissimilarity of two samples using the quantitative Bray-Curtis index^13^,^52^. To examine whether there is a significant difference of functional gene composition between oil phase and water phase, we used three different complementary non-parametric analyses for multivariate data: multi-response permutation procedure (MRPP) (McCune, B. *et al.* Analysis of ecological communities. MJM Software Design: Gleneden Beach, OR, 2002), analysis of similarities (ANOSIM)^53^, and permutational multivariate analysis of variance using distance matrices (Adonis)^54^. Euclidean distance was used to calculate the distance matrix for MRPP, ANOSIM and Adonis analyses. All three methods are based on dissimilarities among samples and their rank order to calculate test statistics. The Monte Carlo permutation was used to test the significance of statistics^55^. All three procedures (MRPP, ANOSIM and Adonis) were performed using the Vegan package v 2.0–4 (Oksanen, J. *et al.* Package Vegan: Community ecology package, version 2.0–4, 2012) in R v. 2.14.0 (RDC Team, 2011).

For pyrosequencing analysis, we removed the low-quality reads shorter than 150 base pairs, with mismatched primer and barcode and with more than one ambiguous nucleotide within correct barcodes or primers. Then we processed and analyzed the data as previously described^56^, and compared them with the Bacterial SILVA database (SILVA version 106, 2011, http://www.arb-silva.de/documentation/release-106/). The OTU was defined by 97% similarity^56^. Venn diagram was used to reflect the bacterial community relationship among production waters, crude oils and injection water. To reveal the diversity and distribution of *Pseudomonas* in oil field samples, we constructed phylogenetic trees based on partial 16S rRNA gene sequences of representative *Pseudomonas* OTUs from this study and *Pseudomonas* type species from RDP (Ribosomal Database Project, 1998, http://rdp.cme.msu.edu/) using the MEGA program, version 5.0^57^.

We calculated the diversity indices including richness, Shannon–Weiner index and Reciprocal of Simpson’s index on the basis of OTUs or functional genes. To understand association between microbial community and geochemical characteristics, we performed canonical correspondence analysis (CCA) to examine whether the geochemical characteristics impacted the microbial communities based on the abundance of OTUs or functional genes, using the Vegan package v 2.0–4 in R v. 2.14.0 (RDC Team, 2011), respectively. To examine overall patterns of variation among the samples, we did hierarchical cluster analysis of functional gene abundance (i.e. normalized signal intensity from GeoChip analysis) or OTU numbers using the Pearson distance method and the complete linkage clustering algorithm with CLUSTER (Gene Cluster 3.0, 2002, http://rana.stanford.edu), and visualized the results in TREEVIEW (TreeView software, 2002, http://rana.stanford.edu/)^51^.

## Additional Information

**How to cite this article**: Cai, M. *et al.* Crude oil as a microbial seed bank with unexpected functional potentials. *Sci. Rep.* **5**, 16057; doi: 10.1038/srep16057 (2015).

The GeoChip data of all samples have been deposited in GEO repository of NCBI under the accession number GSE55293, and the pyrosequencing sequences of all samples have been deposited in NCBI Sequencing Read Archive under the accession number SRP037706.

## Supplementary Material

Supplementary Information
