# Supplementary material for: Crude oil as a microbial seed bank with unexpected functional potentials
Source: Sci Rep. 2015 Nov 3;5:16057. doi: 10.1038/srep16057 (PMC4630613; doi:10.1038/srep16057)
Supplement: Supplementary Information [file srep16057-s1.pdf]

Supplementary Information

Title of the manuscript:

Crude oil as a microbial seed bank with unexpected functional potentials

Author list:

Man Cai[1, +], Yong Nie[1, +], Chang-Qiao Chi[1], Yue-Qin Tang[2], Yan Li[1], Xing-Biao Wang[1], Ze-Shen Liu[1], Yunfeng Yang[3], Jizhong Zhou[3], Xiao-Lei Wu[1, *]

- **Supplementary Methods**

Pyrosequencing and the data analysis

The purified crude DNA was used to amplify the bacterial hypervariable V3 region of the 16S rRNA gene to construct a community library by using tag pyrosequencing. The bar-coded broadly conserved primers 340F and 533R, containing the 454 Life Sciences sequencing adaptors A and B, were used to amplify this region. The forward primer (B-340F) had a 30-nucleotide B adaptor sequence (CCATCTCATCCCTGCGTGTCTCCGACTCAG), and a 10-nucleotide multiplex identifier barcode sequence and 16S rRNA gene primer sequence (TCCTACGGGAGGCAGCAG) as its 3′-end. The reverse primer (A-533R) had the A adaptor sequence (CCTATCCCCTGTGTGCCTTGGCAGTCTCAG), and a 10-nucleotide barcode and 16S rRNA gene primer sequence (TTACCGCGGCTGCTGGCAC) as its 3′-end. The length of the amplicon, including the barcode and 454 primers, was 273 nucleotides. The PCRs were performed in triplicate using 20 μL reactions that contained 0.1 μM of each primer, 20 ng of template DNA, 1× PCR reaction buffer, and 0.02 units/μL Phusion™ High-Fidelity

DNA Polymerase (New England Biolabs, United Kingdom). The amplification conditions consisted of an initial denaturation at 98 ℃ for 2 min, 25 cycles of denaturation at 98 ℃ for 10 s, annealing at 55 ℃ for 10 s, and extension at 72 ℃ for 15 s, and a final extension at 72 ℃ for 10 min. Replicate PCR products from the same samples were assembled in a PCR tube and purified using AxyPrep DNA Gel Extraction Kit (Axygen, China), according to the manufacturer's instructions. Before sequencing, the DNA concentration of each PCR product was determined using Quant-iT PicoGreen double-stranded DNA assay (Invitrogen, Germany). The quality of all the samples was confirmed using an Agilent 2100 bioanalyzer (Agilent, USA). Following quantitation, the amplicons from each reaction mixture were pooled at equimolar ratios and subjected to emulsion PCR to generate amplicon libraries, as recommended by 454 Life Sciences. Amplicon pyrosequencing was then performed from the A end using a 454/Roche A sequencing primer kit on the Roche Genome Sequencer GS FLX Titanium platform at Major Bio Bio-Pharm Technology Co., Ltd., Shanghai, China.

- **Supplementary Figure**

Fig. S1. Venn diagram showing the distribution of OTUs.

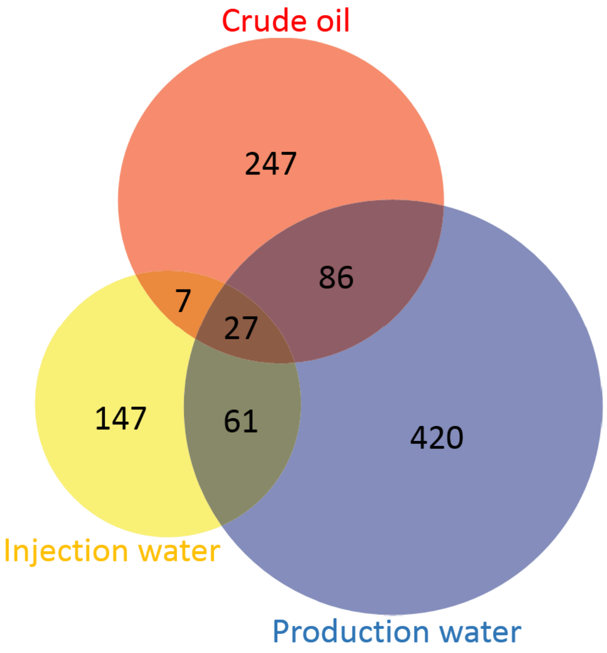

- **Supplementary Tables**

Table S1. **The basal characters of oil production mixture water phase and injection water.**

Sample	Ion Concentration (mg/L)							pH
	Na^+	K^+	Mg^{2+}	Ca^{2+}	Cl^-	SO_4^{2-}	TDS	
**W813**	36340.14	399.38	620.22	2677.44	57451.00	281.85	97770.04	7.47
**W516**	11983.52	102.15	406.84	196.92	20327.10	153.73	33169.29	7.94
**W48**	22456.53	141.19	593.80	544.29	38832.70	ND	62568.51	7.85
**W27**	17310.98	90.48	1463.33	2330.90	34649.87	3.93	55849.49	7.58
**W517**	26086.24	181.09	336.49	1457.26	43822.49	206.27	72036.35	7.56

NO_3^- and NO_2^- were not detected in this study.
Abbreviations: TDS, total dissolved solid (total ion concentration/salinity including Na^+, K^+, Mg^{2+}, Ca^{2+}, Cl^- and SO_4^{2-}); ND, not detected.

Table S2. The character of production wells and relative content of four components. Aliphatic hydrocarbons (ALH), aromatic hydrocarbons (ARH), polar fraction with heteroatoms nitrogen, sulfur and oxygen (NSO) and asphaltenes (ASP) and elements sulfur, nitrogen, carbon and hydrogen in crude oil phase.

Sample	T[1] (℃)	Depth[2] (m)	Relative content (%)							
			ALH	ARH	NSO	ASP	S	N	C	H
**O813**	69.0	2237	65.86	15.86	8.97	9.31	0.380	0.178	67.57	12.13
**O516**	49.5	1073	62.42	17.97	14.71	4.90	0.316	0.286	85.20	12.93
**O48**	41.4	995	60.54	18.71	14.63	6.12	0.375	0.344	85.66	12.72
**O27**	35.5	866	61.45	25.98	10.06	2.51	0.382	0.295	86.20	12.41

1, bottom hole temperature of production wells; 2, production depth of production wells

Table S3. Diversity indices of overall microbial functional genes in all samples.

	O813	O516	O48	O27	W813	W516	W48	W27	W517
Richness[1]	22955	26209	21142	22877	20952	24454	14745	22151	24008
H[2]	9.603	9.722	9.543	9.615	9.396	9.612	9.010	9.589	9.585
1/D[3]	8265.25	9521.46	8963.75	9575.44	5802.58	7802.95	3540.92	8514.01	7780.88
Evenness[4]	0.956	0.956	0.958	0.958	0.944	0.951	0.939	0.958	0.950

[1] Detected valid values number; [2] Shannon–Weiner index; [3] Reciprocal of Simpson’s index; [4] Pielou (Shannon) evenness.

Table S4. Significance of the difference to overall microbial community structure in each production well sample by beta-diversity.

Beta-diversity	O516	O48	O27	W813	W516	W48	W27
**O813**	0.199	0.231	0.297	0.389	0.241	0.450	0.291
**O516**		0.276	0.244	0.372	0.206	0.465	0.239
**O48**			0.319	0.435	0.316	0.467	0.345
**O27**				0.425	0.308	0.526	0.278
**W813**					0.325	0.399	0.304
**W516**						0.446	0.257
**W48**							0.437

Table S5. Significance tests of differences of overall microbial community taxonomic and functional gene compositions between oil and water phase samples.

Statistical approaches	functional gene compositions		taxonomic compositions	
	statistic	P-value	statistic	P-value
MRPP[1]	166.771	0.024	0.2436	0.037
ANOSIM[2]	0.312	0.032	1	0.033
Adonis[3]	0.26	0.001	0.778	0.025

[1]Multi-response permutation procedure, a nonparametric procedure that does not depend on assumptions such as normally distributed data or homogeneous variances, but rather depends on the internal variability of the data.

[2] Analysis of similarities.

[3] Non-parametric multivariate analysis of variance (adonis) using distance matrices.

Table S6. Distribution of functional genes (relative abundance of signal intensity) detected for major metabolic processes among crude oil and water phases samples.

Gene category	O813	O516	O48	O27	Mean[1]	SD[2]	W813	W516	W48	W27	Mean[3]	SD[4]	W517
Aromatics	21.1921	21.1923	21.4953	21.6555	21.3838	0.2307	20.0998	20.9636	19.0450	20.7563	20.2162	0.8632	20.8282
Pesticides related compound	0.8097	0.8022	0.7705	0.7812	0.7909	0.0182	0.6401	0.7277	0.5886	0.7411	0.6744	0.0726	0.7577
Other Hydrocarbons	1.1434	1.1518	1.1169	1.1454	1.1394	0.0154	1.1602	1.1936	1.0398	1.1067	1.1251	0.0672	1.1598
Other organic compound	0.9774	0.9974	1.0756	1.1331	1.0459	0.072	1.1520	1.0194	1.2366	1.0643	1.1181	0.0963	1.0119
Herbicides related compound	2.4911	2.5189	2.6306	2.5401	2.5452	0.0604	2.3933	2.4533	1.9924	2.4283	2.3168	0.2177	2.3865
Chlorinated solvents	0.8346	0.9294	0.82	0.8727	0.8642	0.0488	0.9542	1.0944	0.8643	1.0526	0.9914	0.1031	0.9694
Stress	22.2479	22.1949	22.1112	21.8895	22.1109	0.1579	21.5306	22.1180	21.6163	21.8373	21.7755	0.2623	22.8463
Antibiotic resistance	4.2852	4.1295	3.892	3.7191	4.0065	0.2507	4.3404	4.3166	4.6398	4.3258	4.4057	0.1564	3.9843
Carbon cycling	12.6198	12.8815	13.0578	13.0681	12.9068	0.2096	12.5000	12.2154	12.6361	12.7607	12.5281	0.2341	12.5103
Metal Resistance	12.9147	12.9016	12.8999	13.0302	12.9366	0.0627	13.1702	13.1244	13.4955	12.9686	13.1897	0.2214	13.0740
Nitrogen	7.6364	7.6264	7.4027	7.5428	7.5521	0.1081	8.6915	7.9884	9.2250	7.8835	8.4471	0.6306	8.0557
Sulfur	3.1777	3.175	3.1645	3.0789	3.149	0.0471	3.3840	3.1086	3.5465	3.1490	3.2970	0.2059	3.0640
Other category	1.9789	1.9273	2.0548	2.0646	2.0064	0.0652	1.9533	2.0519	2.0768	2.0585	2.0351	0.0556	1.9544
Virulence	4.0953	4.003	3.9496	3.9915	4.0099	0.0614	4.0807	4.0040	4.1471	4.1040	4.0839	0.0600	3.9195
Bacteria phage	0.4533	0.4695	0.457	0.4717	0.4629	0.0091	0.6325	0.5093	0.5281	0.5675	0.5593	0.0545	0.4502
Energy process	1.3344	1.3969	1.3369	1.3	1.3421	0.0403	1.4591	1.4598	1.4971	1.4952	1.4778	0.0212	1.2926
Phosphorus	1.8082	1.7023	1.7647	1.7157	1.7477	0.0484	1.8580	1.6515	1.8251	1.7008	1.7589	0.0985	1.7351

1, mean of relative abundance of signal intensity from samples O813, O516, O48 and O27; 2, standard deviations of relative abundance of signal intensity from samples O813, O516, O48 and O27; 3, mean of relative abundance of signal intensity from samples W813, W516, W48 and W27; 4, standard deviations of relative abundance of signal intensity from samples W813, W516, W48 and W27.

Table S7. The 99 organic remediation genes which were increased in relative abundance of signal intensity when the temperature/depth increased in four crude oil phase samples. The bold indicates the genes *nahA*, *HBH*, *PobA* and *hmgA*.

Genbank ID	Gene name	Sub-category1	Sub-category2	Organism	O813	O516	O48	O27
108803396	*mdlA*	Aromatics	Aromatic alpha hydroxy acid	*Rubrobacter xylanophilus* DSM 9941	0.6551	0.6504	0.5912	0.5568
118711202	*mdlA*	Aromatics	Aromatic alpha hydroxy acid	*Burkholderia cenocepacia* MC0-3	8.8336	8.7576	7.7846	7.682
148251653	*mdlA*	Aromatics	Aromatic alpha hydroxy acid	*Bradyrhizobium* sp. BTAi1	0.9627	0.7512	0.6366	0.4415
160936953	*mdlA*	Aromatics	Aromatic alpha hydroxy acid	*Clostridium bolteae* ATCC BAA-613	1.3979	1.264	0.7538	0.5112
62468050	*bco*	Aromatics	Aromatic carboxylic acid	uncultured bacterium	0.2928	0.2223	0	0
150257300	*bco*	Aromatics	Aromatic carboxylic acid	*Victivallis vadensis* ATCC BAA-548	0.8346	0.795	0.6641	0.3051
62468078	*bco*	Aromatics	Aromatic carboxylic acid	uncultured bacterium	0.6645	0.3784	0.3251	0
116508772	*GCoADH*	Aromatics	Aromatic carboxylic acid	*Coprinopsis cinerea* okayama7#130	1.031	0.7928	0.7252	0.5975
219679683	*GCoADH*	Aromatics	Aromatic carboxylic acid	*gamma proteobacterium* NOR51-B	0.7605	0.7576	0	0
38524456	*HcaB*	Aromatics	Aromatic carboxylic acid	*Rhodococcus opacus*	0.7398	0.7231	0.5836	0.4213
118686284	***hmgA***	Aromatics	Aromatic carboxylic acid	*Marinomonas* sp. MWYL1	0.6614	0.573	0.5448	0.277
196256716	***hmgA***	Aromatics	Aromatic carboxylic acid	*Cyanothece* sp. PCC 7822	0.1581	0.1498	0	0
197776825	***hmgA***	Aromatics	Aromatic carboxylic acid	*Streptomyces pristinaespiralis* ATCC 25486	0.3944	0.3182	0.3004	0.2304
171095740	*hmgB*	Aromatics	Aromatic carboxylic acid	*Burkholderia ambifaria* MEX-5	0.3968	0.2987	0.2849	0.2803
56677321	*hmgC*	Aromatics	Aromatic carboxylic acid	*Silicibacter pomeroyi* DSS-3	0.5535	0.5125	0.4339	0.3926
214045439	*hmgC*	Aromatics	Aromatic carboxylic acid	*Roseobacter* sp. GAI101	0.5224	0.4782	0.4144	0.2791
91801739	*mdlC*	Aromatics	Aromatic carboxylic acid	*Nitrobacter hamburgensis* X14	1.432	1.0838	0.9855	0.7904
126202187	*mdlC*	Aromatics	Aromatic carboxylic acid	*Pseudomonas stutzeri*	0.9013	0.5344	0.4672	0.3167
46137629	*nagG*	Aromatics	Aromatic carboxylic acid	*Gibberella zeae* PH-1	0.5671	0.4595	0.4219	0.3456
86742884	*nagG*	Aromatics	Aromatic carboxylic acid	*Frankia* sp. CcI3	1.0653	0.9381	0.6195	0.5695
154293167	*nagG*	Aromatics	Aromatic carboxylic acid	*Botryotinia fuckeliana* B05.10	0.3093	0.2086	0	0
161395047	*nagG*	Aromatics	Aromatic carboxylic acid	*Roseobacter litoralis* Och 149	0.7921	0.715	0.604	0.4203

163855735	*nagG*	Aromatics	Aromatic carboxylic acid	*Bordetella petrii* DSM 12804	0.3136	0.2584	0.166	0.1646
84320843	*nagI*	Aromatics	Aromatic carboxylic acid	*Pseudomonas aeruginosa* C3719	1.6222	1.1206	0.9319	0.8214
113529320	*nagL*	Aromatics	Aromatic carboxylic acid	*Ralstonia eutropha* H16	1.0146	0.7537	0.6477	0.5899
148553819	*ohbAB*	Aromatics	Aromatic carboxylic acid	*Sphingomonas wittichii* RW1	0.547	0.5308	0.4753	0.4344
91779734	*PhaB*	Aromatics	Aromatic carboxylic acid	*Burkholderia xenovorans* LB400	2.4929	2.4164	1.4925	0.5612
94314042	*PhaB*	Aromatics	Aromatic carboxylic acid	*Ralstonia metallidurans* CH34	0.6671	0.5187	0.4842	0.4146
20520730	*pimF*	Aromatics	Aromatic carboxylic acid	*Streptomyces coelicolor* A3(2)	0.9404	0.5675	0.5603	0
73542239	*pimF*	Aromatics	Aromatic carboxylic acid	*Ralstonia eutropha* JMP134	2.4686	2.2113	2.0874	1.8941
86138268	*pimF*	Aromatics	Aromatic carboxylic acid	*Roseobacter* sp. MED193	0.5137	0.4649	0.4317	0.421
111224155	*pimF*	Aromatics	Aromatic carboxylic acid	*Frankia alni* ACN14a	1.8495	1.5747	1.4078	1.4051
118617498	*pimF*	Aromatics	Aromatic carboxylic acid	*Mycobacterium ulcerans* Agy99	0.7247	0.5137	0.4213	0.2943
119448330	*pimF*	Aromatics	Aromatic carboxylic acid	marine *gamma proteobacterium* HTCC2143	1.0426	0.8008	0.6369	0.519
119671772	*pimF*	Aromatics	Aromatic carboxylic acid	*Azoarcus* sp. BH72	1.1882	1.0668	0.6093	0.5766
145214770	*pimF*	Aromatics	Aromatic carboxylic acid	*Mycobacterium gilvum* PYR-GCK	1.2122	1.0573	0.9734	0.3907
146307613	*pimF*	Aromatics	Aromatic carboxylic acid	*Pseudomonas mendocina* ymp	4.9591	3.5977	2.7929	2.6692
163259205	*pimF*	Aromatics	Aromatic carboxylic acid	*Bordetella petrii*	1.2948	1.1332	0.8267	0.8008
167587598	*pimF*	Aromatics	Aromatic carboxylic acid	*Burkholderia ubonensis* Bu	1.2376	1.1175	0.5421	0.4887
154246624	***PobA***	Aromatics	Aromatic carboxylic acid	*Xanthobacter autotrophicus* Py2	1.4536	0.9649	0.8781	0.6584
187726416	***PobA***	Aromatics	Aromatic carboxylic acid	*Ralstonia pickettii* 12J	0.557	0.5482	0.339	0.318
84664410	*POBMO*	Aromatics	Aromatic carboxylic acid	*Rhodobacterales* bacterium HTCC2654	0.3284	0.2952	0.2198	0.1268
124260228	*akbF*	Aromatics	BTEX and related aromatics	*Methylibium petroleiphilum* PM1	0.4571	0.3073	0.2972	0.2208
183981521	*akbF*	Aromatics	BTEX and related aromatics	*Mycobacterium marinum* M	1.3582	1.32	0.8271	0.5866
66575509	*catB*	Aromatics	BTEX and related aromatics	*Xanthomonas campestris* pv. *campestris* str. 8004	1.7261	1.1861	1.1556	0.9247
222080905	*catB*	Aromatics	BTEX and related aromatics	*Agrobacterium radiobacter* K84	1.5767	1.2849	1.1991	1.0533
78045925	***HBH***	Aromatics	BTEX and related aromatics	*Xanthomonas campestris* pv. *vesicatoria* str. 85-10	0.3375	0.3228	0	0
74053816	*todC*	Aromatics	BTEX and related aromatics	uncultured bacterium	0.429	0.4259	0.2478	0.2035

218283333	*tutFDG*	Aromatics	BTEX and related aromatics	*Eubacterium biforme* DSM 3989	0.9739	0.6859	0.5961	0.3813
194350066	*xylC*	Aromatics	BTEX and related aromatics	*Stenotrophomonas maltophilia* R551-3	0.8728	0.8072	0.646	0.4801
111223613	*tfdA*	Aromatics	Chlorinated aromatics	*Frankia alni* ACN14a	0.4013	0.3991	0.2628	0.2098
118763178	*tfdA*	Aromatics	Chlorinated aromatics	*Sphingomonas wittichii* RW1	1.5565	1.3591	1.1412	1.0186
134136812	*tfdA*	Aromatics	Chlorinated aromatics	*Burkholderia vietnamiensis* G4	1.5422	1.509	1.2677	1.1422
167348190	*tfdA*	Aromatics	Chlorinated aromatics	*Caulobacter* sp. K31	1.8419	1.7705	1.5128	1.3772
197721534	*carA*	Aromatics	Heterocyclic aromatics	*Streptomyces pristinaespiralis* ATCC 25486	0.6543	0.5287	0.4802	0.4741
145221603	*nhh*	Aromatics	Nitoaromatics	*Mycobacterium gilvum* PYR-GCK	0.791	0.7159	0.502	0.2956
17741838	*nmoA*	Aromatics	Nitoaromatics	*Agrobacterium tumefaciens* str. C58	0.2016	0.1978	0.1761	0.1631
27355865	*nmoA*	Aromatics	Nitoaromatics	*Bradyrhizobium japonicum* USDA 110	0.3027	0.2782	0.2077	0.1999
68200092	*nmoA*	Aromatics	Nitoaromatics	*Frankia* sp. EAN1pec	1.4548	1.2081	0.8898	0.8339
77456479	*nmoA*	Aromatics	Nitoaromatics	*Pseudomonas fluorescens* PfO-1	0.9542	0.7431	0.6322	0.5712
86281200	*nmoA*	Aromatics	Nitoaromatics	*Rhizobium etli* CFN 42	0.982	0.6843	0.6393	0.4252
114329084	*nmoA*	Aromatics	Nitoaromatics	*Granulibacter bethesdensis* CGDNIH1	0.3994	0.2627	0.1906	0.1488
221728375	*nmoA*	Aromatics	Nitoaromatics	*Agrobacterium radiobacter* K84	1.493	1.4613	0.9265	0.8871
88789107	*Arylest*	Aromatics	Other aromatics	*Nitrococcus mobilis* Nb-231	0.4712	0.4146	0.3401	0.3348
218672727	*Arylest*	Aromatics	Other aromatics	*Rhizobium etli* GR56	0.9968	0.9546	0.6661	0.551
22087697	*Catechol*	Aromatics	Other aromatics	uncultured bacterium	4.7704	3.7168	3.0336	2.9558
42627732	*Catechol*	Aromatics	Other aromatics	*Arthrobacter* sp. BA-5-17	12.0271	11.8382	7.8327	0
84043037	*Catechol*	Aromatics	Other aromatics	uncultured bacterium	0.5412	0.4373	0.3819	0.3298
146410979	*Catechol*	Aromatics	Other aromatics	*Bradyrhizobium* sp. BTAi1	0.5277	0.3737	0.3324	0.32
162707916	*Catechol*	Aromatics	Other aromatics	uncultured bacterium	0.2551	0.2515	0	0
87331876	*cumA*	Aromatics	Other aromatics	uncultured bacterium	0.708	0.6477	0.6268	0.5715
78698389	*mdlD*	Aromatics	Other aromatics	*Bradyrhizobium* sp. BTAi1	0.4679	0.445	0	0
212529588	*nitA*	Aromatics	Other aromatics	*Penicillium marneffei* ATCC 18224	1.3775	0.9248	0.895	0.8271
82714858	*pcaG*	Aromatics	Other aromatics	*Pseudomonas putida* F1	0.6824	0.5908	0.4818	0.4477

83723511	*pcaG*	Aromatics	Other aromatics	*Ralstonia solanacearum* UW551	0.6318	0.4994	0.3617	0.3042
84366046	*pcaG*	Aromatics	Other aromatics	*Xanthomonas oryzae* pv. *oryzae* MAFF 311018	0.5344	0.463	0.4013	0.3618
186472723	*pcaG*	Aromatics	Other aromatics	*Burkholderia phymatum* STM815	1.6974	1.6758	1.0595	0.4478
73541393	*pheA*	Aromatics	Other aromatics	*Ralstonia eutropha* JMP134	0.5481	0.529	0.454	0.3944
148565483	*bphA*	Aromatics	Polycyclic aromatics	uncultured bacterium	0.2662	0.2529	0	0
146275509	***nahA***	Aromatics	Polycyclic aromatics	*Novosphingobium aromaticivorans* DSM 12444	2.1985	1.7307	1.4701	1.3694
218548839	***nahA***	Aromatics	Polycyclic aromatics	*Escherichia fergusonii* ATCC 35469	0.6178	0.3491	0.2879	0.2569
219563160	***nahA***	Aromatics	Polycyclic aromatics	uncultured bacterium	1.5132	1.1555	0.9864	0.6644
170721567	*phdCI*	Aromatics	Polycyclic aromatics	*Pseudomonas putida* W619	0.6951	0.5928	0.5669	0.5533
153889031	*dehH109*	Chlorinated solvents		*Ralstonia pickettii* 12D	0.6395	0.5212	0.4949	0.304
124258444	*exaA*	Chlorinated solvents		*Methylibium petroleiphilum* PM1	0.4322	0.3005	0	0
104780666	*atzA*	Herbicides related compound		*Solibacter usitatus* Ellin6076	0.8083	0.7864	0.748	0.742
160942023	*atzA*	Herbicides related compound		*Parvibaculum lavamentivorans* DS-1	0.7012	0.6245	0.5809	0
77383659	*atzB*	Herbicides related compound		*Sorangium cellulosum* 'So ce 56'	2.0706	1.5301	1.2374	0.8707
154246680	*atzB*	Herbicides related compound		*Vibrio vulnificus* YJ016	0.7342	0.6736	0.5702	0.4293
110667993	*trzA*	Herbicides related compound		*Salmonella enterica* subsp. *enterica serovar* Dublin str. CT_02021853	0.9229	0.7532	0.6614	0.5927
116227479	*trzN*	Herbicides related compound		*Rhodococcus* sp. YYL	0.9797	0.75	0.5102	0.4111
154156259	*alkH*	Other Hydrocarbons		*Pseudomonas entomophila* L48	0.3676	0.291	0.2703	0.2124
161163802	*chnB*	Other Hydrocarbons		*Corynebacterium jeikeium* K411	0.7918	0.6783	0.487	0.3566
37679237	*alkK*	Others		*Clostridium bolteae* ATCC BAA-613	0.2349	0.1873	0	0
68264736	*alkK*	Others		*Pseudomonas fluorescens* PfO-1	0.6319	0.5743	0.4673	0.2724
134097783	*alkK*	Others		*Xanthobacter autotrophicus* Py2	0.3307	0.3078	0.2448	0.2419
221535518	*alkK*	Others		*Haloquadratum walsbyi* DSM 16790	0.3792	0.2938	0.2592	0.2453
198243036	*dmsA*	Others		*Saccharopolyspora erythraea* NRRL 2338	0.6114	0.351	0.3166	0.3056
193888337	*thmAB*	Others		*Rhodobacteraceae* bacterium KLH11	0.7893	0.7223	0.4936	0.4915

Table S8. The 133 stress response genes which were increased in relative abundance of signal intensity when the temperature/depth increased in four crude oil phase samples. The bold indicates the gene *dnaK*.

Genbank ID	Gene name	Sub-category	Organism	O813	O516	O48	O27
5020007	***dnaK***	Heat shock	*Rhodothermus marinus*	0.9575	0.9329	0.7181	0.6675
120609913	***dnaK***	Heat shock	*Acidovorax avenae* subsp. *citrulli* AAC00-1	0.5367	0.5345	0.5050	0.3800
84701593	*groEL*	Heat shock	*Parvularcula bermudensis* HTCC2503	0.4859	0.4594	0.3883	0.3520
89331212	*groEL*	Heat shock	*Chlamydophila felis* Fe/C-56	0.5269	0.3637	0	0
226319232	*grpE*	Heat shock	*Deinococcus deserti* VCD115	0.7388	0.6396	0.6047	0.4271
84687811	*glnA*	Nitrogen limitation	*Rhodobacterales* bacterium HTCC2654	1.5663	1.5181	1.5101	0.5617
117928303	*glnA*	Nitrogen limitation	*Acidothermus cellulolyticus* 11B	1.2015	0.8601	0.7483	0.5156
158318237	*glnA*	Nitrogen limitation	*Frankia* sp. EAN1pec	1.2507	1.0139	0.7966	0.4800
146305382	*glnA*	Nitrogen limitation	*Pseudomonas mendocina* ymp	14.8883	9.5251	0	0
163742273	*glnA*	Nitrogen limitation	*Phaeobacter gallaeciensis* 2.10	0.5388	0.5325	0.5090	0.3923
227980740	*glnA*	Nitrogen limitation	*Tsukamurella paurometabola* DSM 20162	0.3166	0.2813	0	0
229749626	*glnA*	Nitrogen limitation	*Stackebrandtia nassauensis* DSM 44728	0.6914	0.6858	0.6309	0.5580
238794434	*proV*	Osmotic stress	*Yersinia intermedia* ATCC 29909	5.0004	3.2297	2.0663	1.1059
134099498	*cydB*	Oxygen limitation	*Saccharopolyspora erythraea* NRRL 2338	0.3934	0.3418	0.2771	0.2103
153803981	*cydB*	Oxygen limitation	*Campylobacter hominis* ATCC BAA-381	0.4243	0.3758	0.3001	0
77995717	*cydB*	Oxygen limitation	*Carboxydothermus hydrogenoformans* Z-2901	0.7870	0.7478	0.5765	0.4745
261877037	*narH*	Oxygen limitation	*Selenomonas noxia* ATCC 43541	0.3867	0.3158	0.3142	0.1133
157919989	*narI*	Oxygen limitation	*Caldivirga maquilingensis* IC-167	1.5455	1.5119	0.8032	0.7563
254470698	*narI*	Oxygen limitation	*Pseudovibrio* sp. JE062	1.2118	1.2002	0.8362	0.5049
224824908	*narJ*	Oxygen limitation	*Lutiella nitroferrum* 2002	1.4416	1.0608	0.8899	0.6264
87284373	*ahpC*	Oxygen stress	*Synechococcus* sp. WH 5701	3.9857	2.7494	2.1790	1.8179
148844004	*ahpC*	Oxygen stress	*Planctomyces maris* DSM 8797	1.2846	1.2163	0.8513	0.3893

152981345	*ahpC*	Oxygen stress	*Janthinobacterium* sp. Marseille	0.5586	0.5419	0.3871	0.3553
152992873	*ahpC*	Oxygen stress	*Sulfurovum* sp. NBC37-1	0.4921	0.3598	0.3128	0.2738
229875748	*ahpC*	Oxygen stress	*Sphaerobacter thermophilus* DSM 20745	1.2632	1.1938	0.8222	0.7609
71909130	*ahpF*	Oxygen stress	*Dechloromonas aromatica* RCB	0.7007	0.4105	0.3930	0.3394
255280719	*ahpF*	Oxygen stress	*Bryantella formatexigens* DSM 14469	1.0790	0.7802	0.6406	0.3854
239908499	*fnr*	Oxygen stress	*Desulfovibrio magneticus* RS-1	0.9022	0.7527	0.6139	0.5668
254473921	*fnr*	Oxygen stress	*Pseudovibrio* sp. JE062	0.5704	0.4543	0.3862	0.3219
260464131	*fnr*	Oxygen stress	*Mesorhizobium opportunistum* WSM2075	0.4270	0.3348	0.2497	0.2019
84690480	*fnr*	Oxygen stress	*Parvularcula bermudensis* HTCC2503	0.6807	0.5318	0.5295	0.4834
86571047	*fnr*	Oxygen stress	*Rhodopseudomonas palustris* HaA2	0.9686	0.8336	0.8033	0.6410
108804637	*fnr*	Oxygen stress	*Rubrobacter xylanophilus* DSM 9941	0.6422	0.5500	0.5386	0.4230
118589376	*fnr*	Oxygen stress	*Stappia aggregata* IAM 12614	0.4243	0.3329	0.2719	0
121606102	*fnr*	Oxygen stress	*Polaromonas naphthalenivorans* CJ2	0.2831	0.2808	0	0
145572032	*fnr*	Oxygen stress	*Pseudomonas stutzeri* A1501	2.9885	2.1565	2.1293	1.8578
229748081	*fnr*	Oxygen stress	*Stackebrandtia nassauensis* DSM 44728	1.0664	0.8949	0.7349	0.5414
261337613	*fnr*	Oxygen stress	*Bifidobacterium gallicum* DSM 20093	1.4588	0.9629	0.9379	0.3416
84509725	*fnr*	Oxygen stress	*Loktanella vestfoldensis* SKA53	2.9774	2.8060	2.3089	2.0460
91682293	*fnr*	Oxygen stress	*Rhodopseudomonas palustris* BisB5	0.5165	0.4563	0.4199	0.2176
144898235	*fnr*	Oxygen stress	*Magnetospirillum gryphiswaldense* MSR-1	0.2758	0.2281	0	0
159897826	*fnr*	Oxygen stress	*Herpetosiphon aurantiacus* ATCC 23779	7.0968	5.3393	4.8125	4.4399
196259193	*fnr*	Oxygen stress	*Cyanothece* sp. PCC 7822	2.5047	2.3522	1.9580	1.4481
239820644	*fnr*	Oxygen stress	*Variovorax paradoxus* S110	0.6927	0.6560	0.6384	0.5526
259417626	*fnr*	Oxygen stress	*Silicibacter* sp. TrichCH4B	0.8845	0.8465	0.7360	0.6064
247547018	*katE*	Oxygen stress	*Paenibacillus* sp. JDR-2	0.3647	0.3300	0.2917	0
256775425	*katE*	Oxygen stress	*Streptomyces hygroscopicus* ATCC 53653	1.4050	0.8692	0.8117	0.1800
116098892	*katE*	Oxygen stress	*Lactobacillus brevis* ATCC 367	0.7230	0.6856	0.5146	0.2051

145300894	*katE*	Oxygen stress	*Aeromonas salmonicida* subsp. *salmonicida* A449	1.1435	1.0694	0.7967	0.2899
229492146	*katE*	Oxygen stress	*Rhodococcus erythropolis* SK121	0.4611	0.2904	0.2596	0.2130
85708160	*oxyR*	Oxygen stress	*Erythrobacter* sp. NAP1	0.9758	0.5099	0.4870	0.4455
227491350	*oxyR*	Oxygen stress	*Corynebacterium pseudogenitalium* ATCC 33035	1.4305	1.1997	1.0536	0.8350
145557579	*oxyR*	Oxygen stress	*Rhodobacter sphaeroides* ATCC 17025	0.4802	0.4218	0.4104	0.4026
213964605	*oxyR*	Oxygen stress	*Corynebacterium amycolatum* SK46	0.4478	0.3903	0.2832	0.1455
229322263	*oxyR*	Oxygen stress	*Rhodococcus erythropolis* SK121	0.7101	0.5169	0.5163	0.5045
56381078	*phoA*	Phosphate limitation	*Geobacillus kaustophilus* HTA426	0.5736	0.5166	0.3505	0.2453
91697353	*phoB*	Phosphate limitation	*Polaromonas* sp. JS666	0.6179	0.3735	0.3225	0.3101
255267734	*phoB*	Phosphate limitation	*Bryantella formatexigens* DSM 14469	0.4281	0.3150	0.2576	0.2047
56313182	*phoB*	Phosphate limitation	*Aromatoleum aromaticum* EbN1	0.4961	0.4867	0.4738	0.4606
151559990	*phoB*	Phosphate limitation	*Ochrobactrum anthropi* ATCC 49188	0.5222	0.4874	0.3449	0.3445
226187406	*pstA*	Phosphate limitation	*Rhodococcus erythropolis* PR4	1.2102	0.9821	0.7380	0.6019
189419948	*pstA*	Phosphate limitation	*Geobacter lovleyi* SZ	0.7948	0.6781	0.6779	0.2031
220907765	*pstA*	Phosphate limitation	*Cyanothece* sp. PCC 7425	0.8533	0.4732	0.4440	0.3719
91788125	*pstA*	Phosphate limitation	*Polaromonas* sp. JS666	0.2913	0.1765	0	0
95113354	*pstA*	Phosphate limitation	*Pseudomonas entomophila* L48	1.4922	1.3726	0.7988	0.7152
158107623	*pstA*	Phosphate limitation	*Frankia* sp. EAN1pec	2.8144	1.5225	1.0443	1.0316
167350934	*pstA*	Phosphate limitation	*Caulobacter* sp. K31	3.3052	2.1786	1.6768	1.6592
269306418	*pstA*	Phosphate limitation	*Xylanimonas cellulosilytica* DSM 15894	1.3133	1.2490	0.9658	0.9350
88790089	*pstB*	Phosphate limitation	*Nitrococcus mobilis* Nb-231	3.0663	0.1652	0	0
145282461	*pstB*	Phosphate limitation	*Pyrobaculum arsenaticum* DSM 13514	0.6224	0.4657	0.4587	0.4209
167350933	*pstB*	Phosphate limitation	*Caulobacter* sp. K31	1.2753	1.1618	0.8978	0.8565
167750376	*pstB*	Phosphate limitation	*Eubacterium siraeum* DSM 15702	0.8503	0.6671	0.3946	0.1993
258594430	*pstB*	Phosphate limitation	*Desulfurivibrio alkaliphilus* AHT2	0.3284	0.2847	0	0
55231571	*pstB*	Phosphate limitation	*Haloarcula marismortui* ATCC 43049	0.4374	0.4284	0.4023	0.3728

116753935	*pstB*	Phosphate limitation	*Methanosaeta thermophila* PT	0.5930	0.5105	0.4982	0.2771
153003295	*pstB*	Phosphate limitation	*Anaeromyxobacter* sp. Fw109-5	9.1453	8.8095	7.7959	6.6441
154250853	*pstB*	Phosphate limitation	*Parvibaculum lavamentivorans* DS-1	0.4560	0.4345	0.3616	0.3189
165926171	*pstB*	Phosphate limitation	*Yersinia pestis biovar Orientalis* str. F1991016	0.8616	0.6682	0.5918	0.5883
239906660	*pstB*	Phosphate limitation	*Desulfovibrio magneticus* RS-1	1.0254	0.8410	0.8382	0.5016
89344130	*pstC*	Phosphate limitation	*Rhodoferax ferrireducens* T118	10.4750	6.6381	4.3611	2.1820
255274115	*pstC*	Phosphate limitation	*Hyphomicrobium denitrificans* ATCC 51888	0.7886	0.6266	0.5157	0.4405
38200740	*pstC*	Phosphate limitation	*Corynebacterium diphtheriae*	1.6944	1.0491	0.8255	0.5034
119897682	*pstC*	Phosphate limitation	*Azoarcus* sp. BH72	0.5836	0.5176	0.5011	0.4274
251808900	*pstC*	Phosphate limitation	*Gallionella ferruginea* ES-2	0.5683	0.4982	0.4562	0.3710
255297134	*pstC*	Phosphate limitation	*Corynebacterium tuberculostearicum* SK141	0.4672	0.4227	0.3962	0.3172
268317380	*pstC*	Phosphate limitation	*Rhodothermus marinus* DSM 4252	0.8259	0.6977	0.5998	0.3803
34102245	*pstC*	Phosphate limitation	*Chromobacterium violaceum* ATCC 12472	0.6539	0.6246	0.4056	0.3647
53758371	*pstC*	Phosphate limitation	*Methylococcus capsulatus* str. Bath	0.4999	0.3213	0	0
77454720	*pstC*	Phosphate limitation	*Rhodococcus erythropolis* PR4	0.8631	0.6056	0.5735	0.5310
161390319	*pstC*	Phosphate limitation	*Phaeobacter gallaeciensis* BS107	0.4772	0.2627	0	0
226245609	*pstC*	Phosphate limitation	*Rhodococcus opacus* B4	0.6502	0.6112	0.5334	0.3877
157368261	*pstS*	Phosphate limitation	*Serratia proteamaculans* 568	1.4585	1.2432	1.0876	0.7456
184192106	*pstS*	Phosphate limitation	*Burkholderia phymatum* STM815	0.3629	0.3432	0.3372	0.3067
158449062	*clpC*	Protein stress	*Coprococcus eutactus* ATCC 27759	12.8862	2.6345	1.2800	1.0272
86553911	*clpC*	Protein stress	*Synechococcus* sp. JA-3-3Ab	1.4609	1.3466	1.2527	1.1493
88802493	*clpC*	Protein stress	*Polaribacter irgensii* 23-P	0.7381	0.5576	0.5026	0.3706
223890914	*clpC*	Protein stress	bacterium Ellin514	0.9260	0.6102	0.5798	0.2867
67920889	*clpC*	Protein stress	*Crocosphaera watsonii* WH 8501	0.8940	0.7466	0.6648	0.6503
68262963	*clpC*	Protein stress	*Corynebacterium jeikeium* K411	0.5158	0.4068	0.3552	0.3159
118618973	*obgE*	Radiation stress	*Mycobacterium ulcerans* Agy99	2.7568	2.3215	1.9199	1.6957

210159308	*obgE*	Radiation stress	*Collinsella stercoris* DSM 13279	3.0311	2.5259	1.9469	1.8068
224604147	*obgE*	Radiation stress	*Lutiella nitroferrum* 2002	0.5088	0.4556	0.3937	0.3586
221230244	*obgE*	Radiation stress	*Mycobacterium leprae* Br4923	0.5153	0.4109	0.3519	0.1977
221563634	*obgE*	Radiation stress	*Geobacter* sp. FRC-32	14.4008	10.2296	7.6564	0.3215
256008154	*obgE*	Radiation stress	*Acidimicrobium ferrooxidans* DSM 10331	1.0121	0.8374	0.6143	0.3567
261209776	*obgE*	Radiation stress	*Vibrio* sp. RC341	0.8670	0.7174	0.6257	0.4285
78035272	*obgE*	Radiation stress	*Xanthomonas campestris pv. vesicatoria* str. 85-10	0.6219	0.5921	0.4372	0.3847
110286770	*obgE*	Radiation stress	*Chelativorans* sp. BNC1	1.2290	0.7662	0.6011	0.5540
124516409	*obgE*	Radiation stress	*Leptospirillum rubarum*	0.7694	0.6738	0.6055	0.2940
260635772	*obgE*	Radiation stress	*Prevotella* sp. oral taxon 472 str. F0295	0.5874	0.5626	0.4389	0.4014
71164188	*sigma_24*	Sigma factors	*Xylella fastidiosa* Dixon	9.0144	7.8726	7.7442	7.1997
227341553	*sigma_24*	Sigma factors	*Rhizobium* sp. NGR234	2.8681	2.5105	2.3696	1.6974
256781200	*sigma_24*	Sigma factors	*Streptomyces hygroscopicus* ATCC 53653	0.9655	0.8069	0.5162	0.4279
261412766	*sigma_24*	Sigma factors	*Aggregatibacter actinomycetemcomitans* D11S-1	1.6432	1.4755	1.0692	0.9627
145574778	*sigma_24*	Sigma factors	*Pseudomonas mendocina* ymp	14.9707	11.5734	0	0
83637289	*sigma_24*	Sigma factors	*Hahella chejuensis* KCTC 2396	0.4713	0.3964	0.3922	0.2174
166712801	*sigma_24*	Sigma factors	*Xanthomonas oryzae* pv. oryzicola BLS256	1.6392	1.0191	0.9861	0.9785
182415495	*sigma_24*	Sigma factors	*Opitutus terrae* PB90-1	0.9718	0.9693	0.9248	0.6762
196229458	*sigma_24*	Sigma factors	*Chthoniobacter flavus* Ellin428	0.3363	0.3231	0.3045	0.2732
217976478	*sigma_24*	Sigma factors	*Methylocella silvestris* BL2	0.8077	0.8019	0.7146	0.4162
223938104	*sigma_24*	Sigma factors	bacterium Ellin514	0.9208	0.8325	0.6877	0.4583
226229027	*sigma_24*	Sigma factors	*Gemmatimonas aurantiaca* T-27	0.3526	0.2245	0	0
254951454	*sigma_24*	Sigma factors	*Dyadobacter fermentans* DSM 18053	0.5046	0.4081	0.3917	0.3575
281424870	*sigma_24*	Sigma factors	*Prevotella oris* F0302	0.7217	0.6622	0.5426	0.3868
158425877	*sigma_32*	Sigma factors	*Azorhizobium caulinodans* ORS 571	0.4989	0.3885	0.3699	0.3645
114797392	*sigma_32*	Sigma factors	*Hyphomonas neptunium* ATCC 15444	0.9511	0.8173	0.7718	0.3882

95134900	*sigma_70*	Sigma factors	*Desulfuromonas acetoxidans* DSM 684	1.1599	0.5934	0.5547	0.2358
258638949	*sigma_70*	Sigma factors	*Pantoea* sp. At-9b	0.6386	0.6065	0.5981	0.3701
87284064	*sigma_70*	Sigma factors	*Synechococcus* sp. WH 5701	0.6364	0.5718	0.4906	0.2457
149921691	*sigma_70*	Sigma factors	*Plesiocystis pacifica* SIR-1	0.5607	0.3853	0.3279	0.3239
168700394	*sigma_70*	Sigma factors	*Gemmata obscuriglobus* UQM 2246	2.7974	2.5039	1.9802	0.3687
196156539	*sigma_70*	Sigma factors	*Alteromonas macleodii* 'Deep ecotype'	1.6903	1.3674	1.2557	1.1378
283851053	*sigma_70*	Sigma factors	*Desulfovibrio* sp. FW1012B	0.6131	0.5808	0.4087	0.3621

Table S9. The 75 stress response genes which were decreased in relative abundance of signal intensity when the temperature/depth increased in four crude oil phase samples. The bold indicates the gene *hrcA*.

Genbank ID	Gene name	Sub-category1	Organism	O813	O516	O48	O27
116175452	*grpE*	Heat shock	*Natrinema* sp. J7	0.2617	0.2762	0.2900	0.3439
226941656	*grpE*	Heat shock	*Laribacter hongkongensis* HLHK9	0.3186	0.9426	1.0190	1.6645
227953141	*grpE*	Heat shock	*Meiothermus silvanus* DSM 9946	0.8507	1.8246	2.4286	3.1234
255067063	*grpE*	Heat shock	*Neisseria sicca* ATCC 29256	0.4306	0.6123	0.6223	1.0652
82703538	***hrcA***	Heat shock	Nitrosospira multiformis ATCC 25196	0	0	0.6239	1.3607
126717282	***hrcA***	Heat shock	*Roseobacter* sp. CCS2	0.5502	1.1298	1.2309	1.8889
154246475	***hrcA***	Heat shock	*Xanthobacter autotrophicus* Py2	0.7624	0.8571	0.9028	1.0963
224817263	***hrcA***	Heat shock	*Thioalkalivibrio* sp. K90mix	0.8055	0.9954	1.0035	1.5871
254432815	***hrcA***	Heat shock	*Cyanobium* sp. PCC 7001	0	0.3144	0.3584	0.4809
88789666	*glnA*	Nitrogen limitation	Nitrococcus mobilis Nb-231	0	0	0.1927	0.3248
89095015	*glnA*	Nitrogen limitation	Oceanospirillum sp. MED92	0	0	0.2148	0.2248
95111842	*glnA*	Nitrogen limitation	*Pseudomonas entomophila* L48	0.5209	0.5544	0.6194	0.6255
254453358	*glnA*	Nitrogen limitation	*Octadecabacter antarcticus* 238	1.2291	1.2920	1.4680	1.9481
52352367	*glnA*	Nitrogen limitation	uncultured archaeon GZfos23H7	0.7845	0.7955	1.0184	1.2070
226309963	*proV*	Osmotic stress	*Brevibacillus brevis* NBRC 100599	0.5830	1.2340	2.0322	2.1320
163854761	*cydA*	Oxygen limitation	*Bordetella petrii* DSM 12804	0	0	0.4721	0.7346
110833406	*narH*	Oxygen limitation	*Alcanivorax borkumensis* SK2	3.7702	5.3734	7.8860	8.1386
120555993	*narI*	Oxygen limitation	*Marinobacter aquaeolei* VT8	1.3877	1.4057	1.4501	1.6843
224026540	*ahpC*	Oxygen stress	*Bacteroides coprophilus* DSM 18228	0.2753	0.3212	0.3728	0.3834
227989465	*ahpC*	Oxygen stress	*Meiothermus silvanus* DSM 9946	0.3378	0.4210	0.4890	0.5048
255532816	*ahpC*	Oxygen stress	*Pedobacter heparinus* DSM 2366	0	0	0.1718	0.1958
257062837	*ahpC*	Oxygen stress	*Slackia heliotrinireducens* DSM 20476	0	0	0.2126	0.2714
50285063	*ahpC*	Oxygen stress	*Candida glabrata* CBS138	0	0.1229	0.2139	0.2881

91799710	*fnr*	Oxygen stress	*Nitrobacter hamburgensis* X14	0.7279	0.9937	1.9890	2.0627
119385205	*fnr*	Oxygen stress	*Paracoccus denitrificans* PD1222	0.3361	0.5419	0.7306	0.7551
120324818	*fnr*	Oxygen stress	*Marinobacter aquaeolei* VT8	0.1948	0.1950	0.2482	0.2591
170654302	*fnr*	Oxygen stress	*Methylobacterium radiotolerans* JCM 2831	0.5483	0.5571	0.6017	0.7242
220914954	*fnr*	Oxygen stress	*Methylobacterium nodulans* ORS 2060	0.5256	0.8005	1.3238	1.3608
264673475	*fnr*	Oxygen stress	*Caulobacter segnis* ATCC 21756	0.9433	0.9879	1.0993	1.4767
145556122	*fnr*	Oxygen stress	*Rhodobacter sphaeroides* ATCC 17025	0.1811	0.2811	0.2882	0.4257
209960557	*fnr*	Oxygen stress	*Rhodospirillum centenum* SW	1.0498	1.4232	1.4372	1.9212
227348748	*katA*	Oxygen stress	*Corynebacterium pseudogenitalium* ATCC 33035	1.6702	1.9198	2.1012	2.9015
89096549	*katE*	Oxygen stress	*Bacillus* sp. NRRL B-14911	0.3852	0.4112	0.5334	0.5349
260903946	*katE*	Oxygen stress	*Brevibacterium linens* BL2	0	0.1902	0.3805	0.4680
15890349	*oxyR*	Oxygen stress	*Agrobacterium tumefaciens* str. C58	0	0	0.2801	0.2807
91188684	*oxyR*	Oxygen stress	*Vibrio alginolyticus* 12G01	0	0	0.2823	0.3631
256742468	*oxyR*	Oxygen stress	*Thiomonas intermedia* K12	0.2103	0.3062	0.5710	0.5717
88776762	*oxyR*	Oxygen stress	*Reinekea* sp. MED297	0.2209	0.2885	0.2999	0.3852
182635267	*oxyR*	Oxygen stress	*Beijerinckia indica* subsp. *indica* ATCC 9039	0	0.2744	0.3475	0.5229
218677074	*oxyR*	Oxygen stress	*Vibrio splendidus* LGP32	0.2541	0.4273	0.4558	0.6400
32444431	*phoA*	Phosphate limitation	*Rhodopirellula baltica* SH 1	0	0.1634	0.2120	0.2781
163761274	*pstA*	Phosphate limitation	*Hoeflea phototrophica* DFL-43	0.3748	0.5295	0.6804	0.7060
241763953	*pstA*	Phosphate limitation	*Acidovorax delafieldii* 2AN	0	0	0.4184	0.5514
12054832	*pstA*	Phosphate limitation	*Streptomyces griseus* subsp. *griseus*	0.5110	0.5963	0.6216	0.8044
124485936	*pstB*	Phosphate limitation	*Methanocorpusculum labreanum* Z	0.3239	0.3507	0.6474	0.6720
149190714	*pstB*	Phosphate limitation	*Vibrio shilonii* AK1	0.2643	0.6077	1.2102	1.2401
256583921	*pstB*	Phosphate limitation	*Cryptobacterium curtum* DSM 15641	0.4890	0.5344	0.6406	0.6484
110635447	*pstB*	Phosphate limitation	*Mesorhizobium* sp. BNC1	1.3050	2.3050	2.4470	3.5107
240102930	*pstB*	Phosphate limitation	*Thermococcus gammatolerans* EJ3	0	0.2310	0.3258	0.5384

260420543	*pstB*	Phosphate limitation	*Citreicella* sp. SE45	0.4484	0.5364	0.5847	0.6585
91681825	*pstC*	Phosphate limitation	*Rhodopseudomonas palustris* BisB5	0	0	0.2197	0.2489
171185022	*pstC*	Phosphate limitation	*Thermoproteus neutrophilus* V24Sta	0.3542	0.5388	0.7517	0.7759
262299877	*pstC*	Phosphate limitation	*Acinetobacter radioresistens* SH164	0.3228	0.5540	0.7671	0.7742
254394474	*pstC*	Phosphate limitation	*Streptomyces pristinaespiralis* ATCC 25486	0.5201	0.7846	0.9697	1.1628
255325922	*pstC*	Phosphate limitation	*Corynebacterium tuberculostearicum* SK141	0.2585	0.3799	0.4918	0.9616
259347147	*pstC*	Phosphate limitation	*Silicibacter* sp. TrichCH4B	0.3691	0.4782	0.5263	0.6328
147851925	*pstS*	Phosphate limitation	*Synechococcus* sp. RCC307	0	0.2109	0.2220	0.3666
218244507	*obgE*	Radiation stress	*Thermus aquaticus* Y51MC23	0.5932	0.6012	0.6624	0.8134
225793943	*obgE*	Radiation stress	*Acidobacterium capsulatum* ATCC 51196	0.2756	0.2797	0.2973	0.3801
55773163	*obgE*	Radiation stress	*Thermus thermophilus* HB8	0.7577	1.7952	1.8493	2.8920
87308465	*sigma_24*	Sigma factors	*Blastopirellula marina* DSM 3645	0.3607	0.4976	0.6604	0.6677
168704291	*sigma_24*	Sigma factors	*Gemmata obscuriglobus* UQM 2246	1.3356	1.4799	2.8424	2.9881
226241603	*sigma_24*	Sigma factors	*Rhodococcus opacus* B4	0.2472	0.3089	0.4023	0.4192
256584581	*sigma_24*	Sigma factors	*Saccharomonospora viridis* DSM 43017	0.2434	0.3186	0.4223	0.4394
268316636	*sigma_24*	Sigma factors	*Rhodothermus marinus* DSM 4252	0	0.3135	0.4932	0.5344
149909450	*sigma_32*	Sigma factors	*Moritella* sp. PE36	0.2574	0.2743	0.3045	0.3048
242133325	*sigma_32*	Sigma factors	*Dickeya dadantii* Ech703	0.2593	0.4841	1.3518	1.3983
110286692	*sigma_32*	Sigma factors	*Chelativorans* sp. BNC1	0.4940	0.7460	0.7601	1.1038
46371303	*sigma_38*	Sigma factors	*Azotobacter chroococcum*	0.4058	0.4911	0.5596	0.5671
113954561	*sigma_70*	Sigma factors	*Synechococcus* sp. CC9311	1.4578	1.5447	1.8778	1.8864
212219307	*sigma_70*	Sigma factors	*Coxiella burnetii* CbuK_Q154	0.2086	0.2364	0.3616	0.3618
226318393	*sigma_70*	Sigma factors	*Deinococcus deserti* VCD115	0.6806	0.8674	1.6004	1.6721
227347540	*sigma_70*	Sigma factors	*Corynebacterium pseudogenitalium* ATCC 33035	1.4692	1.5246	3.8181	3.8970
226714321	*sigma_70*	Sigma factors	*Laribacter hongkongensis* HLHK9	0.3405	0.7039	1.0387	1.4648
237686864	*sigma_70*	Sigma factors	*Teredinibacter turnerae* T7901	1.1910	1.5377	1.6652	1.9027

Table S10. **Diversity indices of bacteria communities in all samples.**

Community	No.Sequences	No. OTUs	C coverage (%)	Richness (Chao1)	Shannons index (Chao & Shen)	Simpson index (MLE)
**O813**	6612	181	0.99	358	1.54	1.71
**O516**	6118	152	0.99	249	1.52	1.73
**O48**	6296	186	0.99	358	1.64	1.81
**O27**	4613	173	0.98	326	1.72	1.88
**W813**	3800	138	0.99	219	3.14	10.32
**W516**	3709	246	0.97	407	3.23	8.71
**W48**	2837	135	0.98	285	2.84	5.94
**W27**	3653	232	0.97	452	3.29	7.74
**W517**	4154	242	0.98	366	3.73	16.12

Table S11. **Numbers and percentage of sequences for phyla in all samples from pyrosequencing. Percentage in brackets.**

Phylum	O8-13	O5-16	O4-8	O2-7	W8-13	W5-16	W4-8	W2-7	W5-17
Proteobacteria	6547(99.02)	6058(99.02)	6161(97.86)	4568(99.02)	3675(96.71)	3681(99.26)	2047(72.15)	3260(89.24)	3340(80.41)
Deferribacteres	11(0.17)	1(0.02)	6(0.10)	17(0.37)	10(0.26)	0	329(11.60)	7(0.19)	1(0.02)
Firmicutes	38(0.57)	23(0.38)	71(1.13)	16(0.35)	105(2.76)	16(0.43)	12(0.42)	22(0.60)	59(1.42)
Bacteroidetes	2(0.03)	0	1(0.02)	5(0.11)	2(0.05)	1(0.03)	2(0.07)	336(9.20)	719(17.31)
Spirochaetes	0	0	7(0.11)	1(0.02)	5(0.13)	4(0.11)	119(4.19)	0	2(0.05)
Actinobacteria	6(0.09)	7(0.11)	0	1(0.02)	0	0	1(0.04)	9(0.25)	0
Thermotogae	0	0	4(0.06)	0	0	2(0.05)	10(0.35)	5(0.14)	18(0.43)
Synergistetes	0	18(0.29)	37(0.59)	0	0	2(0.05)	9(0.32)	2(0.05)	2(0.05)
Chloroflexi	0	0	0	0	0	1(0.03)	0	0	0
**Unidentified Bacteria**	8(0.12)	11(0.18)	9(0.14)	5(0.11)	3(0.08)	2(0.05)	308(10.86)	12(0.33)	13(0.31)

Table S12. Percentages of sequences for genera found in each crude oil and water sample.

Phylum/Class	Genus	O813	O516	O48	O27	W813	W516	W48	W27	W517
Alphaproteobacteria	*Paracoccus*	0	0	0	0	0	0	0	0.03	0
	Pannonibacter	0	0	0	0	0	0	0	0.05	0.02
	Bradyrhizobium	0	0.02	0	0	0	0	0	0	0
	Unidentified	0	0	0	0	0	0.03	0	0.03	4.45
	**Total**	**0**	**0.02**	**0**	**0**	**0**	**0.03**	**0**	**0.11**	**4.48**
Betaproteobacteria	*Petrobacter*	0	0	0	0	0	0	0	0.27	0
	Azoarcus	0	0	0	0	0	0	0	0.03	0
	Unidentified	0	0.02	0	0	0	0	0	0.11	0
	**Total**	**0**	**0.02**	**0**	**0**	**0**	**0**	**0**	**0.41**	**0**
Gammaproteobacteria	*Rheinheimera*	0	0	0	0	0	0	0	0.03	0
	Pseudomonas	97.97	98.87	97.59	96.84	0	55.57	3.21	7.86	0.29
	Marinobacterium	0	0	0	0.11	0	0.05	0	4.74	4.84
	Marinobacter	0.18	0.02	0	0.74	31.26	0.32	0	73.12	25.35
	Idiomarina	0	0	0	0	0	0.67	0	0	2.50
	Halothiobacillus	0	0	0	0	0	0	0	0	0.02
	Halomonas	0.41	0	0.03	0.11	0.11	0	0	0.57	41.86
	Erwinia	0	0	0	0	0	0.03	0	0	0
	Acinetobacter	0.08	0.07	0.03	0.04	0	0	0.07	0.05	0
	Unidentified	0	0	0.05	0.02	0	0.43	0	0.27	0.63
	**Total**	**98.64**	**98.95**	**97.70**	**97.85**	**31.37**	**57.08**	**3.28**	**86.64**	**75.49**
Deltaproteobacteria	*Pelobacter*	0	0	0.03	0.02	0	0	0	0	0
	Geoalkalibacter	0	0	0	0.09	0	0	0	0	0.02
	Desulfuromonas	0	0	0	0	0.08	0	0	0	0.02
	Desulfovibrio	0	0	0	0.13	0	0	0	0.08	0

	Desulfovermiculus	0	0	0	0	0	0	0	0	0.05
	Desulfotignum	0	0	0	0	0	0.03	0	0	0
	Desulfocella	0	0	0	0	0	0.08	0	0	0
	Unidentified	0.15	0.03	0.02	0.85	0.00	1.27	0.21	0.05	0.24
	**Total**	**0.15**	**0.03**	**0.05**	**1.08**	**0.08**	**1.38**	**0.21**	**0.14**	**0.34**
Epsilonproteobacteria	*Sulfurospirillum*	0	0	0.08	0.07	0.03	0.05	37.19	0.68	0.05
	Arcobacter	0.23	0	0	0	65.13	40.50	0	0.82	0
	Unidentified	0	0	0.03	0.02	0.11	0.19	31.48	0.38	0
	**Total**	**0.23**	**0**	**0.11**	**0.09**	**65.26**	**40.74**	**68.66**	**1.89**	**0.05**
Proteobacteria	Unidentified	0	0	0	0	0	0.03	0	0.05	0.05
Firmicutes	*Trichococcus*	0	0	0	0	0	0	0	0	0.02
	Staphylococcus	0.02	0	0	0	0	0	0	0	0
	Mahella	0.02	0	0	0	0	0	0	0	0
	Halocella	0	0	0.02	0	0	0	0	0	0
	Halanaerobium	0.03	0	0.02	0.09	0.05	0	0	0.03	0.39
	Dethiosulfatibacter	0.36	0.08	0.17	0.20	0.26	0.16	0.35	0.36	0.63
	Bacillus	0.03	0.02	0	0	0	0.05	0	0.05	0
	Anoxybacillus	0.02	0	0	0	0	0	0	0	0
	Alkalibacter	0	0	0	0.07	0	0	0	0	0
	Acetobacterium	0	0	0.02	0	0	0	0	0	0
	Unidentified	0.11	0.28	0.91	0	2.45	0.22	0.07	0.16	0.39
	**Total**	**0.57**	**0.38**	**1.13**	**0.35**	**2.76**	**0.43**	**0.42**	**0.60**	**1.42**
Deferribacteres	*Flexistipes*	0.17	0	0.08	0.37	0.26	0	11.56	0.19	0.02
	Unidentified	0	0.02	0.02	0	0	0	0.04	0	0
	**Total**	**0.17**	**0.02**	**0.10**	**0.37**	**0.26**	**0**	**11.60**	**0.19**	**0.02**
Bacteroidetes	*Proteiniphilum*	0.02	0	0	0	0	0	0	0	0

	Cytophage	0	0	0	0	0	0	0	8.92	0
	Prevotella	0	0	0.02	0	0	0	0	0	0
	Marinilabilia	0	0	0	0	0	0	0	0.05	0
	Anaerophaga	0	0	0	0.07	0.03	0	0	0.14	0.05
	Unidentified	0.02	0	0	0.04	0.03	0.03	0.07	0.08	17.26
	**Total**	**0.03**	**0**	**0.02**	**0.11**	**0.05**	**0.03**	**0.07**	**9.20**	**17.31**
Actinobacteria	*Propionibacterium*	0.02	0.02	0	0	0	0	0	0	0
	Jonesia	0	0	0	0.02	0	0	0	0	0
	Dietzia	0.02	0	0	0	0	0	0	0.22	0
	Brevibacterium	0	0.02	0	0	0	0	0	0	0
	Unidentified	0.06	0.08	0	0	0	0	0.04	0.03	0
	**Total**	**0.09**	**0.11**	**0**	**0.02**	**0**	**0**	**0.04**	**0.25**	**0**
Thermotogae	*Thermosipho*	0	0	0	0	0	0	0	0	0.02
	Petrotoga	0	0	0.05	0	0	0.03	0.35	0	0
	Kosmotoga	0	0	0	0	0	0.03	0	0	0
	Geotoga	0	0	0.02	0	0	0	0	0.14	0.41
	**Total**	**0**	**0**	**0.06**	**0**	**0**	**0.05**	**0.35**	**0.14**	**0.43**
Synergistetes	*Thermovirga*	0	0.28	0.57	0	0	0.03	0.32	0.05	0.05
	Anaerobaculum	0	0.02	0.02	0	0	0	0	0	0
	Unidentified	0	0	0	0	0	0.03	0	0	0
	**Total**	**0**	**0.29**	**0.59**	**0**	**0**	**0.05**	**0.32**	**0.05**	**0.05**
Spirochaetes	*Spirochaeta*	0	0	0	0.02	0.13	0.05	2.43	0	0.05
	Unidentified	0	0	0.11	0	0	0.05	1.76	0	0
	**Total**	**0**	**0**	**0.11**	**0.02**	**0.13**	**0.11**	**4.19**	**0**	**0.05**
Chloroflexi	Unidentified	0	0	0	0	0	0.03	0	0	0
**Unidentified Bacteria**		0.12	0.18	0.14	0.11	0.08	0.05	10.86	0.33	0.31

Table S13. *Pseudomonas* species strains presented in Fig.4 and their accession numbers of 16S rRNA gene sequences.

Name	Accession number	Name	Accession number
P. abietaniphila ATCC 700689	AJ011504	P. mandelii CIP 105273	AF058286
P. aeruginosa DSM50071	X06684	P. marginalis LMG 2210	Z76663
P. agarici LMG 2112	Z76652	P. marincola KMM 3042	AB301071
P. alcaligenes IAM12411	D84006	P. mediterranea CFBP 5447	AF386080
P. alcaliphila AL15-21	AB030583	P. mendocina NCIB 10541	D84016
P. amygdali LMG 2123	Z76654	P. meridiana CMS 38	AJ537602
P. anguilliseptica NCIMB 1949	X99540	P. migulae CIP 105470	AF074383
P. antarctica CMS 35	AJ537601	P. mohnii IpA-2	AM293567
P. argentinensis CH01	AY691188	P. monteilii CIP 104883	AF064458
P. arsenicoxydans VC-1	FN645213	P. moorei RW10	AM293566
P. asplenii ATCC 23835	AB021397	P. moraviensis CCM 7280	AY970952
P. azotifigens 6H33b	AB189452	P. mosselii CIP 105259	AF072688
P. azotoformans IAM1603	D84009	P. mucidolens IAM12406	D84017
P. balearica SP1402	U26418	P. nitroreducens IAM 1439	AM088473
P. borbori R-20821	AM114527	P. oleovorans IAM 1508	D84018
P. brassicacearum CIP 109457	EU391388	P. oleovorans RS1	DQ842018
P. brassicacearum DBK11	AF100321	P. orientalis CFML 96-170	AF064457
P. brenneri CFML 97-391	AF268968	P. oryzihabitans IAM 1568	D84004
P. caeni HY-14	EU620679	P. otitidis MCC10330	AY953147
P. cannabina CFBP 2341	AJ492827	P. pachastrellae KMM 330	AB125366
P. caricapapayae ATCC33615	D84010	P. palleroniana CFBP 4389	AY091527
P. cedrina CFML 96-198	AF064461	P. panacis CG20106	AY787208
P. cedrina DSM 14938	AJ492830	P. panipatensis Esp-1	EF424401
P. chlororaphis DSM 50083	Z76673	P. parafulva IAM 1541	AB046999
P. chlororaphis DSM 6698	AY509898	P. pelagia CL-AP6	EU888911
P. chlororaphis JF3835	FJ168539	P. peli R-20805	AM114534
P. chlororaphis NCIB 10068	DQ682655	P. pertucinogena IFO 14163	AB021380
P. cichorii LMG 2162	Z76658	P. plecoglossicida FPC951	AB009457
P. citronellolis DSM 50332	Z76659	P. poae DSM 14936	AJ492829
P. congelans DSM 14939	AJ492828	P. pohangensis H3-R18	DQ339144
P. corrugata ATCC29736	D84012	P. proteolytica CMS 64	AJ537603
P. costantinii CFBP 5705	AF374472	P. pseudoalcaligenes LMG 1225	Z76666
P. cremoricolorata IAM 1541	AB060137	P. psychrophila E-3	AB041885
P. cuatrocienegasensis 1N	EU791281	P. psychrotolerans C36	AJ575816
P. delhiensis RLD-1	DQ339153	P. putida IAM 1236	D84020
P. duriflava HR2	EU046271	P. reinekei MT1	AM293565
P. extremaustralis CT14-3	AJ583501	P. resinovorans LMG 2274	Z76668
P. extremorientalis KMM 3447	AF405328	P. rhizosphaerae IH5	AY152673

P. ficuserectae	JCM 2400	AB021378	P. rhodesiae	CIP 104664	AF064459
P. flavescens	B62	U01916	P. sabulinigri	J64	EU143352
P. fluorescens	IAM 12022	D84013	P. salomonii	CFBP 2022	AY091528
P. fragi	ATCC 4973	AF094733	P. savastanoi	ATCC 13522T	AB021402
P. frederiksbergensis	JAJ28	AJ249382	P. segetis	FR1439	AY770691
P. fulva	AJ 2129	AB046996	P. simiae	OLi	AJ936933
P. fuscovaginae	ICMP 5940	FJ483519	P. straminea	IAM1598	D84023
P. gessardii	CIP 105469	AF074384	P. stutzeri	ATCC 17588	AF094748
P. graminis	DSM 11363	Y11150	P. synxantha	IAM12356	D84025
P. grimontii	CFML 97-514	AF268029	P. syringae	NCPPB 281	DQ318866
P. guineae	LMG 24016	AM491810	P. taeanensis	MS-3	FJ424813
P. indica	DSM 14015	AF302795	P. taetrolens	IAM1653	D84027
P. japonica	IAM 15071	AB126621	P. taiwanensis	BCRC 17751	EU103629
P. jessenii	CIP 105274	AF068259	P. thivervalensis	SBK26	AF100323
P. jinjuensis	Pss 26	AF468448	P. tolaasii	LMG 2342	AF255336
P. kilonensis	520-20	AJ292426	P. tremae	CFBP 6111	AJ492826
P. knackmussii	B13	AF039489	P. trivialis	DSM 14937	AJ492831
P. koreensis	Ps 9-14	AF468452	P. umsongensis	Ps 3-10	AF468450
P. libanensis	CIP 105460	AF057645	P. vancouverensis	ATCC 700688	AJ011507
P. lini	CFBP 5737	AY035996	P. veronii	CIP 104663	AF064460
P. lundensis	ATCC 49968	AB021395	P. viridiflava	CECT 458	AY180972
P. lurida	DSM 15835	AJ581999	P. vranovensis	CCM 7279	AY970951
P. lutea	OK2	AY364537	P. xanthomarina	KMM 1447	AB176954
P. luteola	IAM 13000	D84002	P. xiamenensis	C10-2	DQ088664
P. xinjiangensis	S3-3	EU286805			
